# Genomic patterns of transcription–replication interactions in mouse primary B cells

**DOI:** 10.1093/nar/gkac035

**Published:** 2022-01-31

**Authors:** Commodore P St Germain, Hongchang Zhao, Vrishti Sinha, Lionel A Sanz, Frédéric Chédin, Jacqueline H Barlow

**Affiliations:** Department of Microbiology and Molecular Genetics, University of California Davis, One Shields Avenue, Davis, CA 95616, USA; School of Mathematics and Science, Solano Community College, 4000 Suisun Valley Road, Fairfield, CA 94534, USA; Department of Microbiology and Molecular Genetics, University of California Davis, One Shields Avenue, Davis, CA 95616, USA; Department of Microbiology and Molecular Genetics, University of California Davis, One Shields Avenue, Davis, CA 95616, USA; Department of Molecular and Cellular Biology, University of California Davis, One Shields Avenue, Davis, CA 95616, USA; Department of Molecular and Cellular Biology, University of California Davis, One Shields Avenue, Davis, CA 95616, USA; Department of Microbiology and Molecular Genetics, University of California Davis, One Shields Avenue, Davis, CA 95616, USA

## Abstract

Conflicts between transcription and replication machinery are a potent source of replication stress and genome instability; however, no technique currently exists to identify endogenous genomic locations prone to transcription–replication interactions. Here, we report a novel method to identify genomic loci prone to transcription–replication interactions termed transcription–replication immunoprecipitation on nascent DNA sequencing, TRIPn-Seq. TRIPn-Seq employs the sequential immunoprecipitation of RNA polymerase 2 phosphorylated at serine 5 (RNAP2s5) followed by enrichment of nascent DNA previously labeled with bromodeoxyuridine. Using TRIPn-Seq, we mapped 1009 unique transcription–replication interactions (TRIs) in mouse primary B cells characterized by a bimodal pattern of RNAP2s5, bidirectional transcription, an enrichment of RNA:DNA hybrids, and a high probability of forming G-quadruplexes. TRIs are highly enriched at transcription start sites and map to early replicating regions. TRIs exhibit enhanced Replication Protein A association and TRI-associated genes exhibit higher replication fork termination than control transcription start sites, two marks of replication stress. TRIs colocalize with double-strand DNA breaks, are enriched for deletions, and accumulate mutations in tumors. We propose that replication stress at TRIs induces mutations potentially contributing to age-related disease, as well as tumor formation and development.

## INTRODUCTION

In mammals, the DNA replication machinery must accurately duplicate over 6 billion base pairs per cell division. Replication forks navigate through DNA secondary structures, displace and relocate chromatin-associated proteins, and cope with torsional stress all while maintaining processivity and fidelity. Barriers preventing efficient fork progression—termed replication stress—are a potent source of genome instability, a hallmark of cancer (1). A substantial cause of replication stress is transcription, as RNA polymerase (RNAP) must also navigate the same chromatin template while processively transcribing molecules sometimes over 2 Mb long (2–5).

RNA polymerase II (RNAP2) is a large, multi-subunit complex that transcribes all protein coding genes. RNAP2 activity is tightly regulated at individual genes with each stage of transcription—initiation, elongation and termination—coordinated by the recruitment of multiple complexes through the phosphorylation of the carboxy-terminal domain (CTD) repeats. A series of specific and general transcription factors recruit the unphosphorylated RNAP2 to core promoters, a form poised for activity. The cyclin-dependent kinase Cdk7 then phosphorylates RNAP2 at serine 5 (RNAP2s5), allowing the complex to leave the initiation site (6). RNAP2s5 is found in both promoter-proximal paused and elongating complexes throughout the gene body, and interacts with the spliceosome during co-transcriptional splicing (7,8).

Transcription-replication collisions can arise anywhere transcription and replication machineries occur simultaneously on the same DNA template (4). Transcription and replication create single stranded DNA (ssDNA) during template unwinding potentially allowing stable secondary structures such as G-quadruplexes or stem-loops to form in repetitive DNA sequences. Active transcription also stimulates the formation of co-transcriptionally formed RNA:DNA hybrids (R-loops), three-stranded nucleotide structures formed when nascent RNA re-anneals with the DNA template strand behind elongating RNAP2 (9). R-loops and DNA secondary structures can induce replication-associated genome instability, causing fork stalling or collapse (10–12). Stalled forks can potentially resume replication; however, collapsed forks require resolution through various DNA repair pathways (13).

To date, much of the work directly analyzing transcription–replication collisions has been conducted in bacteria, yeast and *in vitro* (14–16). Studies in bacterial systems show that both co-directional and head-on transcription can induce replication fork stalling and collapse, the latter causing more profound consequences (17–24). Transcription-replication collisions can induce mutations in the form of single basepair substitutions (SBS) as well as short insertions and deletions (indels) at promoters and in protein-coding regions (25). Higher expression and longer gene length correlated with increased accumulation of mutations (28). Both codirectional and convergent transcription–replication collisions experienced damage in the form of indels and base substitutions throughout the gene, but were focused in the promoter region (25). RNAP elongation can also pause at sites of DNA damage and at regulatory sequences causing it to backtrack. GreA and GreB promote transcript cleavage to release paused and backtracked RNA polymerase in bacteria, and their loss increases replication fork stalling, genome instability and cell death (26).

Studies in mammalian cells have helped elucidate the role that transcription–replication collisions play in the context of disease. In an episomal system, head-on and codirectional gene transcription both increase plasmid instability and DNA damage in human cell lines (27). Persistent RNAP2 backtracking also induces genome instability in human cells. A recent report found that U2OS and HEK293T cell lines expressing a mutant form of the transcription elongation factor TFIIS that blocks the rescue of RNAP2 ‘trapped’ in a backtracked or paused state—exhibit increased RNAP2 pausing and DNA breaks (28). In transformed and immortalized lymphocytes, it has been proposed but not demonstrated that transcriptional activity also correlates with aphidicolin-induced genome instability at very long, late-replicating genes located in common fragile sites (CFSs) (3). However, recent studies question this hypothesis, and suggest that transcriptionally-mediated changes in genome organization and replication timing may underlie CFS fragility (29–31). Virtually all studies of transcription–replication collisions in mammalian cells have employed artificial DNA constructs or chemical agents, and use immortalized cell lines with abnormal genotypes and dysregulated cellular processes allowing for indefinite cellular division (27,32–34). Though valuable insights have been gained using these systems, the location of endogenous transcription–replication collisions and their impact on genome stability remains largely unexplored in primary mammalian cells. To understand where transcription and replication machineries may come into conflict, we have developed a method to identify genomic locations where transcription and replication co-occur in a defined spatio-temporal window which we term Transcription-Replication-Interaction (TRI) loci.

To define where TRIs naturally arise, we developed Transcription-Replication IP on Nascent DNA coupled with high-throughput sequencing (TRIPn-Seq), a sequential immunoprecipitation (IP) of RNA polymerase II phosphorylated at serine 5 (RNAP2s5) followed by an IP of bromodeoxyuridine (BrdU) labeled nascent DNA. We used mouse wild-type splenic B cells as a model, as naïve B lymphocytes induce a highly regulated wave of transcription and rapid proliferation that can be reliably mimicked *ex vivo* by antigen/cytokine stimulation (35–37). TRIPn-Seq defined the locations of 1009 independent TRI loci in primary mouse B cells which predominantly occur in early replicating regions. TRIs may be a combination of co-directional and convergent transcription–replication collisions as well as sites of RNAP2s5 reloading onto nascent DNA. TRIs are characterized by high levels of bidirectional transcription, RNA:DNA hybrid formation, and are strongly enriched for genetic sequences prone to forming secondary structures. We propose that TRIs represent genomic loci enriched for multiple structures hindering replication fork progression, and these persistent obstructions to replication fork progression leads to fork collapse, DNA break formation and genome instability.

## MATERIALS AND METHODS

### Mice and B cell harvesting

Mouse spleens were isolated from wild-type (WT) mice with a C57BL/6 background. A 70 μm filter was set in a 5 cm petri dish containing 5 ml of cold wash buffer (see all reagent specifics in reagents table below) and the spleens were placed in the filter and gently pushed through with a 5 ml syringe plunger. The cell suspension was transferred to a 50 ml conical tube, an additional 5 ml of wash buffer was added to the petri dish and the remaining material was gently pushed through the filter and transferred to the 50 ml conical. A final 5 ml of wash buffer was used to rinse the petri dish and transfer the remaining cells to the 50 ml conical tube. The 15 ml cell suspension was centrifuged (Sorvall Legend XTR) at 500 g for 5 min and the supernatant was aspirated. The pellet was resuspended in 4 ml ACK lysis buffer by forcefully pipetting 5 times with a 5 ml pipettor and incubating at room temperature (RT) for 4 min and neutralized with 11 ml of wash buffer. Any large visible non-soluble material was carefully removed with a pipette tip. This was centrifuged at 500 g for 5 min. The supernatant was aspirated, and the pellet was resuspended in 925 μl of wash buffer and additional non-soluble material was removed.

To isolate B cells, 75 μl of mouse CD43 Dynabeads per spleen was aliquoted to a FACS tube and combined with 1 mL of wash buffer. This was placed on a magnet for 3 min and the solution was removed and the beads were resuspended in 75 μl of wash buffer per spleen. The 925 μl of cell suspension was added to the beads, capped and placed on a rotator for 20 min at RT. After incubation, an additional 2 ml of wash buffer was added to the tube, and was pulse centrifuged and placed on a magnet for 5 min. The supernatant was transferred to a new 15 ml conical tube and brought to a total volume of 5–10 ml with wash buffer and cell count was obtained using a Bio-Rad Tc10.

### B cell culture

B cells were added to pre-warmed B cell media (see reagent list below) and stimulated to proliferate using final concentrations of 5 μg/ml LPS, 2.5 ng/ml IL-4 and 250 ng/ml anti-CD180. Cells were plated in 5 ml volumes at 150 000 cells/ml in six-well plates and incubated at 37°C. At 70.5 h, cells were resuspended with a 5 ml pipettor to dislodge the B cells from the plate and transferred to a 50 ml conical. An aliquot of cell suspension was taken for counting and samples were placed back into the incubator for 20 min with the cap loose back to 37°C. For BrdU-positive samples, BrdU was added to a final concentration of 10 μM, inverted 4–6 times to mix, then placed back into the incubator with the cap loose for 30 min.

### Cell fixation for chromatin isolation

Cells were removed from the incubator and immediately crosslinked with 0.75% formaldehyde for 2 min at RT, inverting to mix. Cells were quenched with freshly made 1.25 M glycine stock, bringing the solution to 0.125 M glycine, inverting 4–6 times. Cells were washed by immediately centrifuging at 500 g for 5 min, aspirating the supernatant, and resuspending in 50 ml RT 1× PBS. The samples were washed three times and during the third PBS resuspension, the samples were transferred to different conical tubes with the amount of cells that will be used for the ChIP experiment. The samples were again centrifuged at 500 g for 5 min, supernatant was aspirated, and cell pellet was snap frozen in a 100% EtOH/dry ice bath and stored at –80°C for less than 6 weeks.

### TRIPn and RNAP2s5-ChIP Method 1 (replicates 1 and 2)

Each replicate consists of two samples, including a BrdU negative (BrdU–) and a BrdU-positive (BrdU+) sample. This protocol is written for each sample.

#### Bead preparation for IP #1 RNAP2s5

First, 100 μl of protein G beads were washed with 1 ml TBST, placed on a magnet for 3 min, the supernatant was aspirated, and beads were resuspended in 500 μl TBST. Next, 6 μg of RNAP2s5 antibody was added and placed on a rotator for 4–5 h at 4°C to be used later.

#### Chromatin preparation for IP #1

100–140 million cells were thawed at RT for 10 min, centrifuged at (Sorvall Legend XTR) 500 g for 5 min and supernatant was throrougly aspirated. The cell pellet was resuspended in lysis buffer at 100 μl per 7.5 million cells and vortexed to mix. The lysate was transferred to the sonication tubes at 200–300 μl per tube and sonicated on low for 3 cycles at 30 s on, 30 s off at 4°C. This produced a smear of DNA products of sizes primarily ranging from 500 to 2000 bp on a 1.25% TAE agarose gel. Sonicated samples were centrifuged (Eppendorf 5424R) at 13 000 g for 5 min and the supernatant was transferred to a conical tube. For gel samples to visualize fragment lengths, 1 μl gel of sonicated cell solution was added to 19 μl PBS and proteinase K was added to a final concentration of 100 μg/ml and placed in a shaking dry bath at 55°C, for 14 h, at 500 rpm.

#### IP #1- RNAP2s5

The supernatant was diluted 1:10 with TBST, mixed and portions were transferred to an Amicon concentrator tube. This was centrifuged (Sorvall Legend XTR) at 4000 g until the remaining solution in the top chamber was ∼1 ml. This was pipetted to mix and dislodge any material that was bound to the filter surface and additional sonicated chromatin solution was added and centrifuged again. This was repeated until the total amount was reduced to ∼1.5 ml.

The protein G/RNAP2s5 antibody mixture was pulse centrifuged, placed on a magnet for 5 min and the supernatant was aspirated and discarded. The solution in the Amicon filter was pipetted again to mix and dislodge any material bound to the filter and transferred to the 1.5 ml tube that contained the washed protein G/antibody combination. This was placed on a rotator at 4°C O/N.

#### DNA isolation following IP #1

The next day the samples were pulse centrifuged, placed on a magnet for 5 min and the supernatant was discarded. The beads were gently resuspended with a 1000 μl micropipette with 1 ml of low salt buffer, transferred to a new 1.5 ml tube, and placed on a magnet for 5 min. This step was repeated with high salt buffer and then LiCl buffer also transferring to new tubes after each resuspension. The LiCl buffer was discarded and beads were resuspended with 60 μl of elution buffer and placed on a shaking dry bath at 45°C for 20 min at 700 rpm. This was placed on a magnet for 3 min and the supernatant was transferred to a new tube. The beads were rinsed with an additional 60 μl of elution buffer, placed on a magnet for 3 min, and the additional supernatant was added to the previous 60 μl of eluate. This was stored this in 4°C until ready for overnight reverse crosslinking. To reverse crosslink, 280 μl of TBST and 100 μg/ml of proteinase K was added and placed on shaking dry bath at 55°C for 14 h at 500 rpm.

[Specifically for TRIPn, not for RNAP2s5-ChIP: 20 μl of protein G beads were washed with 500 μl TBST, placed on a magnet for 3 min, supernatant was aspirated, and beads were resuspended in 100 uL TBST. 1 μg of anti-BrdU antibody was added and placed on a rotator for 4–5 h at 4 ^o^C to be used later.]

The next day the samples were pulse centrifuged, transferred to sonication tubes and sonicated on high for 14 cycles at 15 s on, 45 s off at 4°C to reduce the fragment size to 200–500 bp. The samples were pulse centrifuged and transferred to a new 1.5 ml tube. 400 μl of phenol/chloroform was added and vortexed on high for 10 s. Samples were pulse centrifuged, transferred to a phase lock tube, and centrifuged again for 5 min at 13 000 g. The top layer was transferred to a new 1.5 ml tube, 1128 of 100% EtOH, 41 ul of 3 M sodium acetate and 2 μl of glycogen were added and vortexed on high for 10 s. This was incubated at –80 °C for 1–2 h, centrifuged (Eppendorf 5424R) for 30 min at 13 000 g at 4°C, the supernatant was discarded, 500 μl of EtOH was added without dissolving the pellet, and centrifuged (Eppendorf 5424R) again for 15 min at 13 000 g at 4°C. The supernatant was discarded, and the pellet was dried until all the EtOH was evaporated—about 15 min and no more than 20 min—by placing the 1.5 ml tube upside down at a 45° degree angle, ensuring the pellet does not slide down the tube. The pellet was resuspended in 52 μl of 0.1× TE for 30 min while briefly vortexing and centrifuging (Eppendorf 5424R) every 10 min. The concentration was measured by Qubit.

#### Adapter liagtion following IP #1

The NEBNext End Prep protocol and reagents were used followed by adapter ligation. Briefly, adapters were diluted 1:10 in 10 mM Tris–HCl pH 8.0/10 mM NaCl. The NEBNext Ultra II DNA Library Prep Kit for Illumina directions were used for an AmpureXP clean-up starting with 86 μl (92% of total volume) AmpureXP beads.

[Specifically for TRIPn, not for RNAP2s5-ChIP]

#### IP #2 for BrdU

The DNA attached to the AmpureXP beads was eluted with 30 μl of water and transferred to a 1.5 ml tube. 120 μl of TBST was added to the tube, heated in a shaking dry bath at 95°C for 5 min and immediately placed on ice.

The protein G/BrdU antibody mixture was pulse centrifuged, placed on a magnet for 5 min and the supernatant was aspirated and discarded. The solution from the AmpureXP clean-up was transferred to the tube that contained the washed protein G / BrdU antibody combination. This was placed on a rotator at 4°C O/N.

#### IP #2 washes

The next day the samples were pulse centrifuged, placed on a magnet for 5 min and the supernatant was aspirated and discarded. The beads were gently resuspended with a 1000 μl micropipette with 500 μl low salt buffer, transferred to a new 1.5 ml tube, and placed on a magnet for 5 min. This step was repeated with high salt buffer and then LiCl buffer also transferring to new tubes after each resuspension. The LiCl buffer was discarded and the beads were resuspended with 25 μl of elution buffer and place on a shaking dry bath at 45°C for 20 min at 700 rpm. This was placed on a magnet for 3 min and the supernatant was transferred to a new tube. The beads were rinsed with an additional 25 μl of elution buffer, placed on a magnet for 3 min, and additional supernatant was added to the previous 25 μl of elution.

#### IP #2 clean-up

50 μl of water was added and a two-step AmpureXP clean-up was performed to remove large and small fragments. 55 μl beads (55% of total volume) was first added, mixed, and incubated for 5 min, and placed on a magnet for 5 min. The supernatant was collected and an additional 25 μl beads was added (80% total Ampure solution) and the rest of the procedure was performed as indicated in the NEB protocol.

#### Library preparation

The DNA bound to the AmpureXP beads was eluted in 32 μl of 0.1X TE. 1 μl was used to perform a test qPCR amplification to ensure an adequate amount of material in the library amplification. 15 μl was stored at –20°C and 15 μl was used for library amplification (this can be stored at –20°C for future amplification). Library amplification was also performed using the NEBNext Ultra II DNA Library Prep Kit for Illumina using NEB Illumina Adaptors and was purified using the Ampure XP beads. 50 μl of water was added to the 50 μl of PCR product and a two-step AmpureXP clean-up was performed the same as above using 58 μl of beads for the first step and 20 μl of beads for the second step and eluted in 17 μl of 0.1× TE. One μl was used for a Qubit measurement for DNA concentration and 1 μl was used for a Bioanalyzer analysis for fragment size. The library was submitted to Novogene for sequencing on an Illumina Hiseq Platform PE150.

### TRIPn and RNAP2s5-ChIP Method 2 (replicate 3)

Each replicate consists of two samples, 1 BrdU– and 1 BrdU+ and this protocol is written for one sample.

#### Chromatin preparation

Ten million cells were thawed at RT for 10 min, centrifuged at 500 g for 5 min and remaining fluid was aspirated. The cell pellet was resuspended in RIPA+ buffer at 300 μl per 10 million cells, vortexed to mix and transferred to sonication tubes at 200–300 μl per tube. Samples were sonicated on high at 4°C for 8 cycles at 15 s on, 45 s off, rotating sonicator positions after 4 cycles. To confirm sonication efficiency, 2 μl gel of sonicated cell solution was added to 18 μl PBS and proteinase K (final concentration 100 μg/ml) and placed in a shaking dry bath at 55°C, for 14 h, at 500 rpm. Successful samples produced a smear of DNA products between 200 and 1500 bp on an 1.25% TAE agarose gel. Sonicated samples were centrifuged at 13 000 g for 5 min and the supernatant was transferred to a 1.5 ml tube.

#### IP #1- RNAP2s5

The supernatant was diluted 1:5 with TBST and 1 μg of RNAP2s5 antibody was added per 275 of diluted chromatin supernatant. This was placed on a rotator at 4°C O/N.

Ten percent of the IP volume of protein G beads were washed with 1000 μl TBST, placed on a magnet for 3 min and the supernatant was discarded. The RNAP2s5 IP solution from the previous night was pulse centrifuged and pipetted into the protein G beads and pipetted to mix. This was placed on a rotator for 4–5 h at 4°C.

Samples were pulse centrifuged, placed on a magnet for 5 min and the supernatant was aspirated and discarded. Beads were gently resuspended with a 1000 μl micropipette with 1 ml of low salt buffer, transferred to a new 1.5 ml tube and placed on a magnet for 5 min. This step was repeated with high salt buffer and then LiCl buffer also transferring to new tubes after each resuspension. The LiCl buffer was discarded and beads were resuspended with 100 ul of elution buffer and place on a shaking dry bath at 45°C for 20 min at 700 rpm. The beads were placed on a magnet for 3 min and supernatant was transferred to a new tube. Beads were washed with an additional 100 μl of elution buffer, placed on a magnet for 3 min, and the supernatant was added to the previous 100 μl of elution. This was stored in 4°C until ready for overnight reverse crosslinking. To reverse crosslink, 200 μl of PBS and 100 μg/ml of proteinase K was added and placed on shaking dry bath at 55 °C for 14 h at 500 rpm.

#### DNA isolation following IP #1

The next day the samples were pulse centrifuged, transferred to sonication tubes and sonicated on high for 10 cycles at 15 s on, 45 s off at 4°C to reduce the fragment size to 200–500 bp. The samples were pulse centrifuged and transferred to a new 1.5 ml tube. 400 μl of phenol/chloroform was added and vortexed on high for 10 s. This was pulse centrifuged, transferred to a phase lock tube, and centrifuged again for 5 min at 13 000 g. The top layer was transferred to a new 1.5 ml tube, 1128 of 100% EtOH, 41 μl of 3M sodium acetate and 2 ul of glycogen was added and vortexed on high for 10 s. This was incubated at –80°C for 1–2 h, centrifuged (Eppendorf 5424R) for 30 min at 13 000 g at 4°C, the supernatant was discarded, 500 ml of EtOH was added without dissolving the pellet, and centrifuged (Eppendorf 5424R) again for 15 min at 13 000 g at 4°C. The supernatant was discarded, and the pellet was dried until all the EtOH was evaporated for 15 but no more than 20 min by placing the 1.5 ml tube upside down at a ∼45° angle. The pellet was resuspended in 52 μl of 0.1× TE for 30 min while briefly vortexing and centrifuging every 10 min. The concentration was measured by Qubit.

#### Adapter liagtion following IP #1

The NEBNext End Prep protocol and reagents were used followed by adapter ligation. Adapters were diluted 1:10 in 10 mM Tris–HCl pH 8.0 and 10 mM NaCl. The NEBNext Ultra II DNA Library Prep Kit for Illumina directions were used for an AmpureXP clean-up starting with 86 μl (92% of total volume) AmpureXP beads.

[Specifically for TRIPn, not for RNAP2s5-ChIP]

#### IP #2 for BrdU

The DNA attached to the AmpureXP beads was eluted with 50 μl of 0.1× TE and transferred to a 1.5 ml tube. 150 μl of TBST was added to the tube, heated in a shaking dry bath at 95°C for 5 min and immediately placed on ice. 1 μg of BrdU antibody was added and placed on a rotator at 4°C overnight.

The next day 20 μl of protein G beads were washed with 500 μl TBST, placed on a magnet for 3 min, supernatant was discarded, and beads were resuspended in 100 μl TBST. The BrdU IP solution from the previous night was pulse centrifuged and pipetted into the protein G beads and pipetted to mix. The protein G-BrdU IP mixture was placed on a rotator for 4–5 h at 4°C.

#### IP #2 washes

After incubation, the samples were pulse centrifuged, placed on a magnet for 5 min and the supernatant was aspirated and discarded. The beads were gently resuspended with a 1000 μl micropipette with 500 μl low salt buffer, transferred to a new 1.5 ml tube, and placed on a magnet for 5 min. This step was repeated with high salt buffer and then LiCl buffer also transferring to new tubes after each resuspension. We discarded the LiCl buffer and resuspended with 25 μl of elution buffer and place on a shaking dry bath at 45°C for 20 min at 700 rpm. This was placed on a magnet for 3 min and the supernatant was transferred to a new tube. The beads were rinsed with an additional 25 μl of elution buffer, placed on a magnet for 3 min, and the additional supernatant was added to the previous 25 μl of elution.

#### IP #2 clean-up

100 μl of water was added and a two-step AmpureXP clean-up was performed to remove large and small fragments. 82.5 μl beads (55% of total volume) was first added, mixed and incubated for 5 min, and placed on a magnet for 5 min. The supernatant was collected and saved and an additional 37.5 μl beads (80% total Ampure solution) was added, and the rest of the procedure was performed as indicated in the NEB protocol.]

Library preparation: DNA bound to the AmpureXP beads was eluted in 32 μl of 0.1× TE. A test qPCR amplification was performed on 1 μl of sample to ensure adequate material for library amplification. The remaining sample was stored in –20°C and 15 μl was used for library amplification (this can be stored at -20°C for future amplification). Library amplification was performed using the NEBNext Ultra II DNA Library Prep Kit for Illumina using NEB Illumina Adaptors and was purified using the Ampure XP beads. Fifty μl of water was added to 50 μl of PCR product and a two-step AmpureXP clean-up was performed the same as above using 58 μl of beads for the first step and 20 μl of beads for the second step and eluted in 17 μl of 0.1× TE. DNA concentration was measured by Qubit on 1 μl of sample, and 1 μl was used for a Bioanalyzer analysis of fragment size. The library was submitted to Novogene for sequencing on an Illumina Hiseq Platform PE150.

### Flow cytometry—replication timing for S phase cells

B cells were isolated and grown as described above. For the control sample, HU was added to a final concentration of 10 μM. At the indicated time points, 2 ml of cells were aliquoted to a 15 ml conical and centrifuged (Eppendorf 5424R) at 500 g for 5 min, the supernatant was aspirated, and the cells were resuspended in 1.5 ml of cold PBS. The cells were permeabilized and fixed by adding 3.5 ml of 4°C, 100% EtOH slowly while mixing and incubating at –20°C for 20 min. The cells were washed twice after centrifugation at 850 g for 5 min, discarding the supernatant, and resuspending in 5 ml of PBS. After the second wash, the cells were resuspended in 2 ml of PBS and propidium iodide (PI) was added to a final concentration of 10 μg/ml. PI incorporation was measured on a BD Canto II, comparing experimental cells to HU treated control cells.

### DNA–RNA immunoprecipitation (DRIP)

DRIP-Seq was performed as described previously (38).

### Reagents

See Table 1.

**Table 1.** Reagents

Reagents/materials	Details
ACK lysis buffer	155 mM ammonium chloride, 10 mM potassium bicarbonate, 0.1 mM EDTA, pH 7.2–7.4, filtered
AmpureXP beads	Fisher Scientific (NC9933872)
Antibodies	Mouse anti-RNA polymerase 2 phospho-serine 5 (RNAP2s5) antibody, (4H8), Abcam# ab5408; BrdU – BD Bioscience – 555627 (3D4); IgG – mouse IgG isotype control – Abcam: 37355
Anti-CD180	Purified rat anti-mouse clone RP-14 (BD Biosciences #552128, lot# 8172646) 0.5 mg/ml
B cell purification beads	Dynabeads untouched CD43 mouse B cell isolation kit (Thermo Fisher, 11422D)
B cell spleen filter	Corning 70 μm filter (#431751)
B cell growth and stimulation media	500 ml RPM1-1640, 50 ml FBS, 96.2 U/ml penicillin/streptomycin, 9.6 mM HEPES, 1.9 mM glutamine, 1.0 mM sodium pyruvate, 53 μM BME
Cell counter	Bio-Rad Tc10
Centrifuges	Sorvall Legend XTR – 75003180 rotor; Eppendorf 5424R – 24 tube rotor
Concentrator tubes	Amicon Ultracel-100 regenerated cellulose membrane, 4 ml sample volume, Millipore Sigma: UFC10008
Elution buffer	1% SDS, 100 mM NaHCO_3_
FBS	Gemini, heat inactivated, LOT# A79EOOG
Formaldehyde	Fisher Scientific 37% formaldehyde (BP531-500)
Glycogen	Amresco N632-0.5 ml, 20 mg/ml
High salt wash buffer	20 mM Tris–HCl pH 8.0, 500 mM NaCl, 2 mM EDTA, 1% triton, 0.1% SDS
IL4	Sigma – I1020, lot# MKCF5055. 5 μg resuspended in 1 ml wash buffer, final concentration used may vary due to batch activity
Library preparation kit	NEBNext Ultra II DNA Library Prep Kit for Illumina; NEBNext Multiplex Oligos for Illumina – Index Primer Set 1
LiCl wash buffer	10 mM Tris–HCl pH 8.0, 0.25 M LiCl, 1 mM EDTA, 1% NP-40, 1% sodium deoxycholate
Low salt wash buffer	20 mM Tris–HCl pH 8.0, 150 mM NaCl, 2 mM EDTA, 1% Triton, 0.1% SDS
LPS	Sigma #L2630 25, lot# 028M4022V, 25 mg resuspended in 1 ml RPMI, final concentration used may vary due to batch activity
Lysis buffer	1% SDS, 50 mM Tris pH 8.0
PBS	137 mM NaCl, 2.7 mM KCl, 10 mM Na_2_PO_4_, 1.8 mM KH_2_PO_4_, filtered
Phase lock tubes	Phase Lock Gel Light, Quanta Bio, VWR# 10847-800
Phenol/chloroform isoamyl alcohol	Fisher Scientific, 25:24:1
Protein G beads	Invitrogen #10004D 30 mg/ml
Proteinase K	20 mg/ml, UC Davis Supply
RIPA	150 mM NaCl, 0.5% Na-deoxycholate, 1% NP-40, 0.1% SDS, 50 mM Tris pH 8.0
RIPA+	300 μl RIPA buffer, 7.5 μl 20% SDS
Sonication tubes	TPX 1.5 microtubes from Cosmo Bio Co., Ltd.
Sonicator	Bioruptor UCD-300 w/ 1.5 eppendorf tubes
TBST	20 mM Tris–HCl pH 8.0, 150 mM NaCl, 0.1% Tween-20
TE	10 mM Tris–HCl 8.0, 1 mM EDTA

## COMPUTATIONAL METHODS AND RESOURCES

### Programs to perform general computational and transformation functions

Bedtools v 2.26.0 (https://bedtools.readthedocs.io/en/latest/) (39); Python v 2.7.15rc1 (https://www.python.org/); Python v 3.6.9 (https://www.python.org/); SciPy v 1.0: matplotlib, pandas, numpy (https://www.scipy.org) (40–44); UCSC table browser (45); UCSC executable programs: wigToBigWig, liftOver, bigWigToBedGraph (http://hgdownload.soe.ucsc.edu/admin/exe/) (46).

### Basic fastq to bigwig workflow

If using downloaded SRA data, fastq-dump 2.7.0 (https://ncbi.github.io/sra-tools/fastq-dump.html) was used to generate the fastq from SRA file (47). Fastq reads were trimmed with Trimmomatic v 0.36 (http://www.usadellab.org/cms/?page = trimmomatic) (48) and aligned to the mm10 mouse genome (49) with Bowtie2 v 2.2.8 only keeping uniquely mapped reads (http://bowtie-bio.sourceforge.net/bowtie2/index.shtml) (50). Unmapped reads, unpaired reads and PCR duplicates were discarded, and the file was converted into BAM format using Samtools v 1.9 (http://www.htslib.org/) (51). Specifically, unmapped reads were removed using samtools view -bh -F 4, unpaired reads were removed using samtools view -bh -f 2, PCR duplicates were removed using samtools fixmate -m and samtools markdup -r. The BAM file was converted to a bedgraph file using bedtools genomecov and normalized by reads per million (RPM). The bedgraph file was converted to a bigwig file and visualized using the UCSC genome browser (https://genome.ucsc.edu/) (52).

### TRI Identification

Peaks were called on three independent biological replicate experimental (BrdU+) and three control (BrdU–) TRIPn-Seq BAM files using MACS2 callpeak v 2.2.5 (https://github.com/macs3-project/MACS) (53) using the parameters: -f BAMPE –nomodel -g mm. The experimental and control counts were then analyzed and compared to each other using R 4.0.2 (https://www.r-project.org/) and DiffBind v 2.16.0 (https://bioconductor.org/packages/release/bioc/html/DiffBind.html) (54) using default parameters except: dba.count(OBJECT, minOverlap = 0) (54). Additional TSS peaks were generated by creating 1 kb windows ±1 kb from the TSSs in sliding increments of 100 bp and each peak set was individually analyzed with the edgeR analysis of DiffBind using the Benjamini-Hochberg algorithm. All peaks that were measured to have differential signal between experimental and control with an FDR ≤0.05 were combined and merged and the highest FDR was kept.

### Overlap/association experiments

Genomic Association Tester v 1.0 (GAT) (https://gat.readthedocs.io/en/latest/) (55) was used to test association between TRIs and TSSs (56) in 1 kb windows, 24 h NT END-seq peaks (57) and CpG islands (58,59). The workspace was restricted to the feature (TSSs, END-seq peaks, CpG) merged with all genes that overlapped with RNAP2s5 ChIP-Seq peaks + 500 bp upstream of the TSS. GAT was also used to test the overlap in kbp between TRIs and early replicating fragile sites (ERFSs) where control regions were restricted to early replicating regions and (60) and common fragile sites (CFSs) where control regions were restricted to late replicating regions (61–66). TimEX data was used to define early and late regions as described in (67,68). Overlaps were represented in bp because of the large size difference.

### TRI TSS orientations

Plus and minus strand TRITSSs that were within 3kb of their respective upstream regions and also intersected with GRO-Seq signal were classified as two divergent TSSs. Plus and minus strand TRITSSs that were within 3kb of their respective downstream regions and intersected with GRO-Seq signal were classified as two convergent TSSs. Plus or minus strand TRITSSs that had no other annotated TSSs within 3 kb but intersected with GRO-Seq on the plus and minus strands were classified as annotated single genes with unannotated divergent transcription. Intersect analyses were performed using Bedtools Intersect.

### Profile plots and heatmaps

Profile plots and heatmaps were created by using deepTools 3.1.3 (https://deeptools.readthedocs.io/en/develop/) plotMatrix, plotProfile and plotHeatmap while removing the gaps and blacklisted regions and plotting the median and standard error. In order to compare the same amount of measurements between the regions of interest, cTSSs were shuffled and a number of regions were plotted to match the same number of TRI or TRITSS regions (69).

### TimEX

DeepTools bamCompare was used to calculate the log_2_ difference between the reads in the activated B cell BAM file and the resting B cell BAM file (GSE116318 (67)) with the following parameters: –operation log2 –smoothLength 500 –binSize 100 –bl mm10.GAP.BLACKLIST.BED –effectiveGenomeSize 2652783500.

### OK-Seq

DeepTools bamCoverage was used to count reads in the forward strand BAM file in 1 kb windows using the following parameters: -bs 1000, –bl mm10.GAP.BLACKLIST.BED –effectiveGenomeSize 2652783500 -of bedgraph. The mm10 genome was partitioned into 1 kb windows. The reads from bamCoverage were mapped to the mm10 genome 1 kb windows using the mean. This was repeated for the reverse strand BAM file. Using the files from the forward and reverse strands, a new replication fork direction (RFD) file in bedgraph format was created using this calculation (R – F) / (R + F) then converted to a bigwig file (70).

### TRI overlap with tumor mutations

The Mouse Tumor Biology Database (71) provided us with data that listed gene names associated with mutations found in sequenced mouse tumors (23 625 mutations) and unique gene names (6525 genes) were extracted. The 1198 TRI genes were compared with the tumor mutation unique gene name list. A complete gene list was constructed by taking all MGI annotations (302 974 annotations) and removing annotations that were predicted genes, TSS only and did not contain precise coordinates or a specific strand and 28 398 genes remained. 1198 random genes were extracted and compared with the tumor mutation gene list. We performed the random extraction and comparison 999 times. For analysis of mutation type, all mutations were kept including non-unique gene names. The same procedure was performed using data from Sleeping Beauty Cancer Driver Database (72) but using 1231 identified cancer driver genes.

### Variant analysis

bcftools v 1.9 (http://samtools.github.io/bcftools/bcftools.html) mpileup was used to create genotype likelihoods on four different whole genome sequencing files GSM3227968, GSM3227969 (67), GSM4098725 and GSM4098729 (73) using the parameters: -B -Ou -f mm10.fa –max-depth 4000 –max-idepth 2000. Next bcftools call was used to identify variant sites using the parameters: mA -Ob. The bcf files were then indexed using bcftools index. The regions of interest used for TRITSSs and cTSSs were ±500 bp from the annotated TSS from the MGI and merged if containing overlapping regions. The regions of interest used for genes were the entire length of the annotated gene +500 bp upstream of the TSS (74). Next, the 4 bcf files were converted to vcf files. The five regions of interest were extracted for each of the four vcf files using bcftools convert with the following parameters: –threads 8 -O z -R [regions of interest] for a total of 20 files. The files were sorted using bcftools sort -O z and indexed using bcftools index. The four TRI files were then combined using bcftools merge -O v and this was repeated for the TRITSS, cTSS, TRI full gene, control full gene files. Mutational signatures were generated using the merged files using SigProfilerMatrixGenerator v 1.1 (https://github.com/AlexandrovLab/SigProfilerMatrixGenerator) (75). To measure enrichment or depletion of single basepair substitutions and indels at TRIs, cTSSs and TSSs that had RNAP2s5 signal within genic regions, we counted the variants within 1 kb regions centered at the 1198 TRITSSs and then randomly sampled 1198 regions of the 12 957 cTSSs and counted the variants and repeated the random sampling for a total of 999 times (76). The same procedure was repeated for TRI genes, cTSS genes and all genes with RNAP2s5 signal except the samples were split into 1 kb windows to normalize for gene length.

### G4 analysis

G4 quadruplex formation was predicted using G4 Hunter v 3.0 in 400 bp windows using the -w 25 -s 1.4 parameters (https://github.com/AnimaTardeb/G4Hunter) (77).

### GC skew

GC skew was calculated by measuring (G – C)/(G + C) in 200 bp regions using bedtools nuc using a sliding window of 1 bp where the result of the calculation corresponds to the center of the 200 bp region (78).

### Motif extraction

Motifs were extracted using HOMER v 4.9.1 findMotifsGenome.pl in 400 bp windows (http://homer.ucsd.edu/homer/) (79).

### Gene enrichment analysis

Gene enrichment analysis was performed using MouseMine (www.mousemine.org) (80), g:Profiler (https://biit.cs.ut.ee/gprofiler/gost) (81) and Enrichr (https://maayanlab.cloud/Enrichr/) (82,83).

## STATISTICAL ANALYSES

Statistical analyses between the profile plot signals were performed similar to the DeepTools method of creating the profile plots. Further normalization was not needed because the comparisons between TRITSSs and cTSSs were from the same already normalized bigwig files. First the bigwigs were converted to bedgraphs, splitting the region of interest (30 kb for TimEX, 5 kb for GC-Skew, 1 kb for RPA ChIP-Seq, GC percent and DNA methylation) into smaller windows (1 kb for TimEX, 10 bp for all others). Next, the the median bedgraph signal intensity was mapped onto the new smaller windows using bedtools map, then the mean signal of values was calculated across the entire region of interest. The difference in the calculated values in the regions of interest between 1198 TRITSSs and 12 957 cTSSs was expressed using a *P*-value calculated using Wilcoxon ranksums in SciPy (84,85). The *P*-values for enrichment and depletion of single basepair substitutions and indels and all overlap analyses was calculated using the empirical *P*-value where *P* = (*r* + 1)/(*n* + 1), where *r* is the number of times the hypothesis tested is false and *n* is the total number of comparisons (55,86). The enrichment or depletion of TRI gene type, MTB mutation type and COSMIC mutation frequency were calculated using the hypergeometric distribution *P*-value (87).

## RESULTS

### Transcription-replication immunoprecipitation on nascent DNA followed by whole-genome sequencing (TRIPn-Seq).

**Figure 1.**
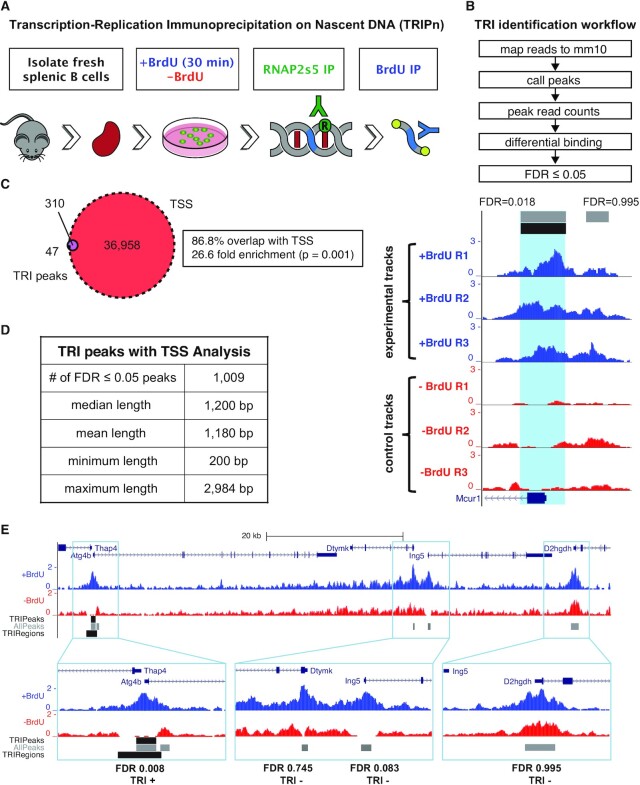
Transcription-Replication ImmunoPrecipitation on Nascent DNA followed by high throughput sequencing (TRIPn-Seq). (**A**) Benchtop workflow of TRIPn-Seq. Mice spleen were extracted, and B cells were isolated and stimulated for growth. Prior to harvesting, BrdU was added to the medium for 30 min. The first IP was for RNAP2s5. Library adapters were added and then the second IP was performed for BrdU. (**B**) Bioinformatic workflow of TRIPn-Seq and representative UCSC genome browser tracks of RPM normalized reads for three TRIPn-Seq experiments (blue) and three controls (red). All experiments are performed in wild-type (WT) mouse B cells (mBCs) unless noted otherwise. Gray bars are areas analyzed along with the calculated FDR by DiffBind and edgeR of differential signal between experimental and control. Black bar and light blue shading are areas that are considered TRIs because the FDR ≤0.05. The black bar with arrows at the bottom shows the Mcur1 gene and direction of transcription. (**C**) Venn diagram showing the overlap between TRIs and transcription start sites (TSS), showing empirical *P*-value. (**D**) Properties of 1009 TRIs including additional analyzed TSSs. (**E**) Representative UCSC genome browser tracks showing TRI positive regions with FDR ≤ 0.05 and TRI negative regions (FDR > 0.05) and their overlap with TSSs. AllPeaks (gray) are all peaks found using MACS2 and analyzed with DiffBind, TRIPeaks (upper black bar) are peaks with differential signal compared to control with an FDR ≤0.05, TRIRegions (lower black bar) also includes analyzed TSSs.

To map TRIs genome-wide, we developed TRIPn-Seq, a sequential IP coupled to genome-wide sequencing that first isolates RNAP2s5-bound DNA, then enriches for nascent DNA (nDNA) labeled with BrdU (Figure 1A). We stimulated freshly-isolated mouse splenic B cells for rapid proliferation for 72 h, then pulse labeled with BrdU for 30 min. Colocalization of RNAP2s5 and nDNA can occur by replication encountering active transcription complexes; however, RNAP2s5 can also reload onto DNA post-replication. To minimize detection of RNAP2s5-BrdU co-IP from post-replication, we transiently labeled DNA for 30 min. Cells were then crosslinked, sonicated to produce chromatin fragments, and subjected to the first IP for RNAP2s5. Transcription and replication utilize very large protein complexes, therefore they may be separated by a significant amount of DNA. Both processes also create superhelical stresses which may induce even further linear separation on the DNA template. Thus, the distance between transcription and replication markers—RNAP2s5 and BrdU—is likely longer than typical ChIP-Seq experiment chromatin fragments which are between 200 and 500 bp. We chose an initial chromatin fragment size of 300–1500 bp to increase isolation of DNA fragments with both nDNA and RNAP2s5, while keeping the fragment size short enough to still retain high spatial resolution. The RNAP2s5-IP chromatin eluted DNA was sonicated again, ligated to sequencing library adapters and used for the second IP for BrdU. Library preparation was completed on the BrdU-IP eluate and subjected to high-throughput sequencing.

To identify genomic areas specifically enriched for both RNAP2s5 and BrdU, we performed TRIPn-Seq in triplicate on cells incubated with BrdU and compared to those without BrdU. Two different ChIP methods (described as Method 1 and Method 2) were employed for TRIPn-Seq because optimization of TRIPn-Seq revealed that different ChIP conditions gave distinct RNAP2s5 ChIP-Seq peak sets. Representative UCSC genome browser tracks show that Method 1 and Method 2 both exhibited strong RNAP2s5 signal at gene promoters, but Method 2 showed additional transcription-associated peaks not found in Method 1 (Supplementary Figure S1A, yellow). Analysis of the three biological replicates showed BrdU-positive experimental and BrdU-negative control samples strongly clustered regardless of ChIP method (Supplementary figure S1B). We mapped the sequencing data from three biological replicates and three controls to the mouse genome assembly mm10 and called peaks. We measured differential binding comparing the peaks pooled from experimental samples against controls and initially found 357 TRI loci with significantly more signal over the control using a false discovery rate (FDR) cutoff of ≤0.05 (Figure 1B, Supplementary Figure S1C).

### TRIPn-Seq signal correlates with active TSSs

Visual inspection of TRI peaks on the UCSC genome browser indicated a strong overlap with transcription start sites (TSSs, Figure 1C). Indeed, 86.8% of TRIs (334/357) overlapped with TSSs curated from Mouse Genome Informatics (MGI), a 26.6-fold enrichment over random chance (56). To define additional TRI peaks near TSSs, we analyzed TRIPn-Seq signal ±1 kb from all TSSs in 800 bp sliding windows with 100 bp steps and each sliding window set was analyzed individually. Loci with FDR ≤0.05 were merged with the original 357 TRI loci for a total of 1009 TRI loci (Figure 1D). We found that TRI experimental peaks with FDR ranging from 0.008 to 0.995 showed similar TRIPn-Seq signals, the difference between low and high FDR was in the difference in signal between the experimental samples and controls, shown in a representative genome browser snapshot (Figure 1E).

To define the number of genes associated with TRIs, we analyzed ±1.5 kb flanking the TRI peak centers and found that some TRIs overlap multiple genes, such that the 1009 TRI loci are associated with 1221 total TRIs when intersecting nearby annotated genes on the plus and minus strands (1198 unique genes, Supplementary Table S1). For controls, we used 12 957 control TSSs (cTSSs) which have RNAP2s5 signal but were not considered TRIs (FDR > 0.05; Figure 1C, E). TRIs do not center precisely at TSSs, therefore we also performed all downstream analyses centering the 1198 TSS-overlapping TRIs on the TSSs (TRITSSs) for more accurate comparison to cTSSs.

### General properties of TRIs and their associated genes

We next compared the length of genes associated with TRIs to all RNAP2s5-bound genes. We found that TRI-associated genes are consistently longer than RNAP2s5-bound genes (median length 41 017 bp for TRIs versus 17 694 bp for cTSS genes; *P* = 3.45 × 10^–65^, Wilcoxon rank sum test). When we restricted analysis to protein coding genes only, the median length of TRI genes was still significantly longer than controls (41 071 bp versus 20 830 bp; *P* = 6.06 × 10^–40^, Wilcoxon rank sum test; Supplementary Figure S1D). We analyzed the type of genes that TRIs overlapped and found an enrichment of protein-coding genes and a depletion of pseudogenes and long non-coding RNA genes compared to all genes (Supplementary Table S2). We also observed a depletion of microRNA (miRNA) genes. This may be an underrepresentation as miRNAs are often found in clusters, and our analyses associated a single miRNA per TRI. Additionally, we found that TRI frequency per chromosome strongly correlated with the number of genes per chromosome. However, there was a depletion of TRIs on ChrX and Chr7 (Supplementary Figure S1E).

**Figure 2.**
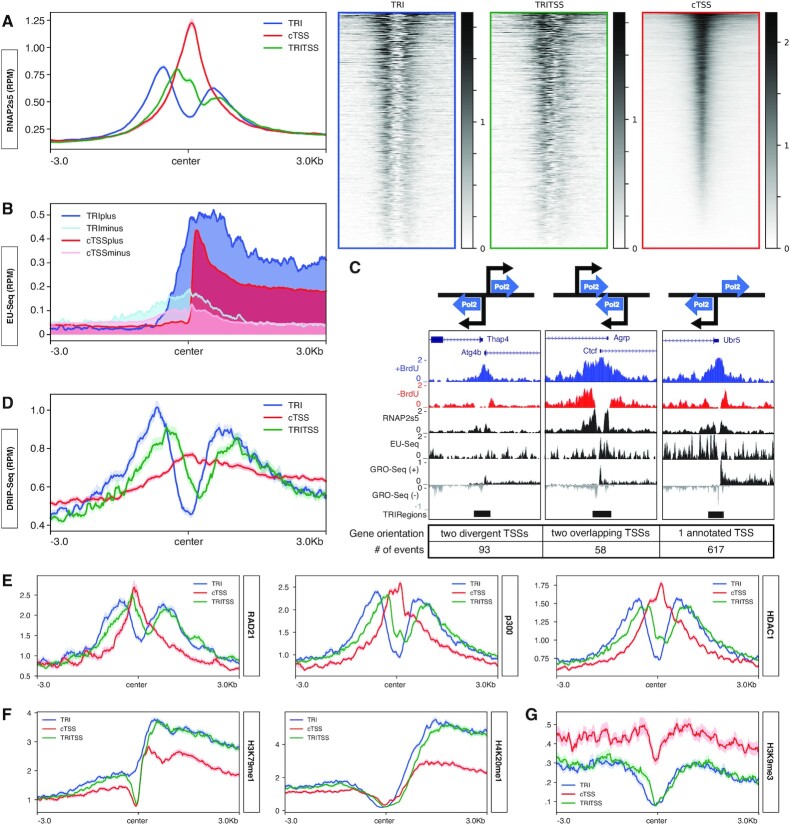
Characterization of transcriptional activity at TRIs. (**A**) Median Method 2 RNAP2s5 ChIP-Seq signal profiles centered near TSSs for TRIs (blue, *n* = 1221), TRITSSs (green, *n* = 1198) and cTSSs (red, *n* = 12 957). Median Method 2 RNAP2s5 ChIP-Seq signal heatmaps centered near TSSs for TRIs (blue, *n* = 1221), TRITSSs (green, *n* = 1198) and cTSSs (red, *n* = 1218). (**B**) Median EU-Seq signal profiles centered near TSSs for plus strand nascent RNA at TRIs (blue), minus strand nascent RNA at TRIs (light blue), plus strand nascent RNA at cTSSs (red) and minus strand nascent RNA at cTSSs (pink). (**C**) Graphic representation of possible RNAP2s5 orientations and representative UCSC genome browser tracks of RPM normalized reads of (from top to bottom) Mouse Genome Informatics gene annotation, TRI region, TRI control, RNAP2s5 ChIP-Seq, EU-Seq and GRO-Seq, and TRI peak for Ctcf, Gm5914, Thap4, Atg4b and Ubr5. (**D**) Median DRIP-Seq signal profiles centered near TSSs for TRIs, TRITSSs and cTSSs. (**E**) Median RAD21, P300 and HDAC1 ChIP-Seq signal profiles in RPKM for TRIs, TRITSSs and cTSSs. (**F**) Median H3K79me1 and H4K20me1 ChIP-Seq signal profiles in RPKM for TRIs, TRITSSs and cTSSs. (**G**) Median H3K9me3 ChIP-Seq signal profile in RPKM for TRIs, TRITSSs and cTSSs. *All profile plot analyses are performed in mBCs. Shaded areas on the unfilled line plots are the standard error.

In addition to identifying unique RNAP2s5 ChIP-Seq peaks, the Method 2 signal pattern was similar to reports of bimodal RNAP2 signals from high-coverage ChIP-Seq experiments and single nucleotide resolution footprinting studies, indicating that Method 2 provides higher resolution for RNAP2s5 signal (88,89). Using the Method 2 RNAP2s5 ChIP-Seq data, we found that cTSSs had a single RNAP2s5 peak in the center while TRIs had two flanking RNAP2s5 signals (Figure 2A, left). TRI peaks were greatly outnumbered by cTSS peaks, potentially confounding signal comparisons along individual loci by metagene analysis. To compare the same number of peaks, cTSSs were randomly shuffled and similar number of regions were plotted for cTSSs and TRIs. Heatmaps showed the majority of TRI/TRITSS loci exhibit bimodal distribution of RNAP2s5 signal, while cTSSs harbored a single central peak (Figure 2A, right). We also measured RNAP2s5 occupancy throughout the entire gene body and at transcription termination sites (TTSs), and found that genes associated with cTSSs had more RNAP2s5 occupancy than TRITSS genes (Supplementary Figure S2A).

### Characterization of transcriptional activity at TRIs

The RNAP2s5 signal distribution at TRIs suggests two populations of RNAP2s5, potentially transcribing in opposing directions. To determine if both RNAP2s5 peaks correlate with RNA production, we analyzed nascent transcription at TRIs and cTSSs using a published EU-Seq dataset (67). We found overlapping plus and minus strand nascent transcription signal at TRIs and cTSSs, with more transcription in both directions along TRIs than cTSSs (Figure 2B). These results indicate TRI genes have more bidirectional transcription producing overlapping and partially antisense RNAs. When evaluating full genes, nascent transcription was higher at TRI genes near the TSS (Supplementary Figure S2B, upper panel). Analysis of nascent transcription using an independent GRO-Seq dataset similarly showed overlapping bidirectional transcription at TRIs (73) (Supplementary Figure S2C, upper panel). TRITSSs exhibited more nascent transcription than cTSSs by EU-Seq, while TRITSS and cTSS had similar levels by GRO-Seq (Supplementary Figure S2B and C, lower panels). This apparent difference is likely due to the types of transcriptional activity measured; EU-Seq measures active RNAP *in vivo* but requires longer labeling times, while GRO-Seq measures transcriptionally-competent (paused and active) RNAP molecules *in vitro* (90,91). Taken together, these results indicate that TRITSSs have more active RNAP2 molecules than cTSSs.

The bimodal distribution of RNAP2s5 surrounding TRIs suggests transcription initiation from two distinct sites in close proximity. Upon analyzing the arrangement of genes within TRI regions, three distinct patterns emerged: two annotated genes with non-overlapping divergent TSSs, divergently-transcribing gene pairs with overlapping genic regions, and annotated single genes with unannotated divergent transcription (Figure 2C). Using GRO-Seq to call peaks for plus and minus strand transcription within 3 kb of TRITSSs, we found that ∼76% of TRI regions contained peaks on both plus and minus strands indicative of bidirectional transcription. Some regions examined also exhibited EU-Seq signal on both plus and minus strands, consistent with GRO-Seq results (Figure 2C).

R-loops are three-stranded nucleotide structures primarily formed co-transcriptionally by nascent RNA hybridizing to template DNA and looping out the non-template DNA strand. DNA:RNA ImmunoPrecipitation (DRIP) using the S9.6 antibody maps R-loops genome-wide in mammalian cells (92). To investigate if TRIs are enriched for R-loops, we next performed DRIP-Seq on stimulated B cells. Similar to RNAPs5 signal, both TRIs and TRITSSs had bimodal DRIP-Seq signal, while cTSSs showed a single central peak of lower amplitude (Figure 2D, Supplementary Figure S2D). Due to the distinct pattern differences, we did not compare relative R-loop levels between TRISSs and cTSSs. We observed higher DRIP-Seq signal along the length of the gene body and at TTSs at cTSS genes than TRI-associated genes, consistent with RNAP2s5 signal (Supplementary Figure S2E). Taken together, the bimodal RNAP2s5 signal and high levels of bimodal DRIP signal at TRIs indicates two distinct populations of active RNAP2 producing RNA molecules with a propensity for template association.

### Epigenetic landscape and chromatin modifier association at TRIs

We next examined the chromatin landscape surrounding TRIs and cTSSs using published ChIP-Seq datasets from mouse primary B cells (73). Like RNAP2s5, the chromatin insulator CTCF and cohesin complex member RAD21 had two flanking peaks of signal at TRIs while cTSS exhibit a single central peak signal (Figure 2E, Supplementary Figure S3A). A similar pattern was observed for the histone acetyltransferases (HAT) p300 and GCN5 (Figure 2E, Supplementary Figure S3A). The increase in HAT occupancy was reflected in histone acetylation, where TRIs exhibited higher H4K12Ac, H4K16Ac and H2BK20Ac signal upstream of the transcription start site relative to cTSSs, as well as histone deacetylases HDAC1 and HDAC2 where there was a single peak in the center of cTSSs and two flanking peaks at TRIs (Figure 2E, Supplementary Figure S3B). These results are consistent with two steady populations of active RNAP2 at TRIs.

To further define the epigenetic state of TRI regions, we next analyzed histone methylation patterns (73) also in stimulated WT mouse primary B cells. Areas flanking TRIs exhibited higher levels of H3K79me1/2, H4K20me1, H3K27me2, H3K4me1/2/3 and H3K9me1—all marks associated with active chromatin (Figure 2F, Supplementary Figure S3C). Further, levels of methylation marks associated with silent or repressed genes—H3K9me2/3 and H3K27me3—were lower than controls (Figure 2G, Supplementary Figure S3D). Together, these results indicate that TRI regions exhibit marks of an active chromatin state (93). Of note, TRIs showed higher flanking signals of H3K79me2 and H4K20me1 than cTSSs (Figure 2F, Supplementary Figure S3C). Both marks are associated with non-homologous end joining (NHEJ)-mediated double-strand break (DSB) repair, possibly suggesting that TRIs accumulate DNA damage (94). However higher H3K79me2 and H4K20me1 densities also correlate with faster elongation rate and gene length (95,96). Thus, the increase of these marks at TRIs may reflect their correlation with longer genes (Supplementary Figure S1D). We conclude the enrichment of chromatin modifying enzymes and histone marks correlating with an active transcriptional state upstream and downstream of TRITSSs is consistent with bidirectional transcription.

### Replication characteristics of TRIs

#### TRI regions are proximal to replication initiation and termination zones

**Figure 3.**
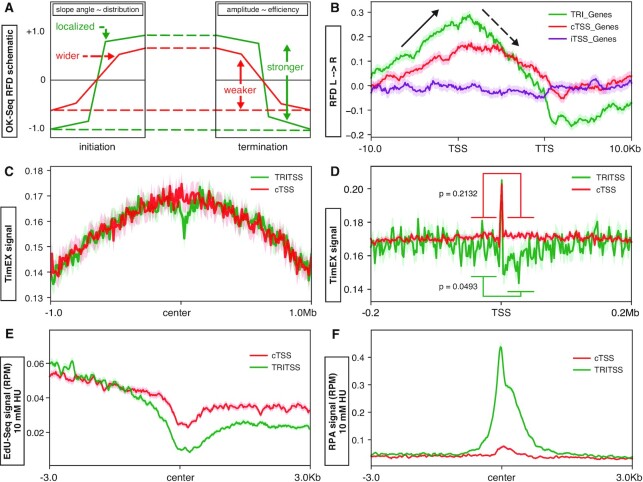
Replication characteristics at TRIs. (**A**) Graphic representation of example of OK-Seq RFD data and interpretation at replication initiation and termination zones: slope correlates with origin localizartion, and amplitude correlates with origin efficiency. (**B**) Median OK-Seq signal profile scaled to full genes ±10 kb of TRI genes (green, *n* = 583), cTSS genes (red, *n* = 6558) and inactive TSS genes (purple, *n* = 11 612). Solid black arrows indicate initiation zones and dashed black arrows indicate termination zones. (**C**) Median TimEX signal profiles centered at TSS ± 1.0 Mb for TRI genes (green) and cTSSs genes (red). (**D**) Median TimEX signal profiles centered at TSS ± 200 Kb for TRI genes (green) and cTSSs genes (red). (**E**) Median EdU-Seq signal profiles on cells treated with 10 mM HU centered at TSSs for TRITSSs and cTSSs. (**F**) Median RPA ChIP-Seq signal profiles on cells treated with 10 mM HU centered at TSSs for TRITSSs and cTSSs For TRIs or TRITSS compared to cTSS, *P* < 1.0 × 10^–250^; Wilcoxon rank sum test). *All profile plot analyses are performed in mBCs. Shaded areas on the unfilled line plots are the standard error.

To assess the replication characteristics of TRIs, we analyzed OK-Seq which maps Okazaki fragments of replication forks and can determine replication initiation and termination zones as well as their efficiency and directionality (70) (schematic of expected OK-Seq profiles, Figure 3A). Analysis of OK-Seq signals surrounding TRI and cTSS genes (including 10 kb upstream and downstream of TTS) revealed a positive replication fork directionality (RFD) slope upstream of TRIs and cTSS genes indicating the promoter regions of both groups are origin-rich, in contrast to non-transcribed inactive TSSs (iTSSs) (Figure 3B, Supplementary Figure S4A, B). These results further support the idea that replication preferentially starts near the transcription start sites of genes (97,98). TRI genes have a steeper positive slope and show higher signal than cTSS genes, indicating that TRI promoter regions tend to contain highly localized and more efficient origins. We also found that OK-Seq had a more negative slope through TRI gene bodies, indicating they experience more termination events than cTSS genes. In a 20 kb window of analysis centered at the TSS, there is a modest dip in RFD signal right at TRITSSs which may indicate these genes contain difficult to replicate areas (Supplementary Figure S4C). These results indicate regions upstream of TRIs are enriched for origins with strong firing efficiency.

#### TRIs occur in early replicating zones

To independently assess the replication timing of TRIs, we next analyzed Timing Express (TimEX) data from mouse primary B cells (98). TimEX measures replication timing by calculating the ratio of DNA copy number of cycling cells to resting cells in G0/G1; a higher ratio indicates more replicated DNA in S phase cells and thus earlier replication (68). Analysis of TimEX signal in a 2 Mb window around TRI and cTSS genes shows high TimEX signal, indicating these are some of the earliest replicating regions in the genome (Figure 3C) (98). This is consistent with prior reports showing high transcription correlates with early replication (99). However we observed a reduction in TRITSS TimEX signal near the center, suggesting this region replicates later. Indeed, in a 0.4 Mb window we found that TimEX signal was reduced at TRITSSs, and this reduction was only observed downstream of the TSSs (Figure 3D; *P* = 0.0493, Wilcoxon rank sum test). Unlike TRIs, the TimEX signal for cTSSs was similar upstream and downstream of the TSS (Figure 3D; *P* = 0.2132, Wilcoxon rank sum test). These results indicate that the region downstream of TRITSSs exhibit delayed replication.

#### Contribution of transcriptional activity to replication timing at TRIs

High transcriptional activity has been associated with high origin density, origin efficiency and earlier replication timing (70). To determine if high transcriptional activity of TRIs can explain their origin enrichment and replication timing, we compared TRIs to cTSSs matched for activity using EU-Seq data. We found that the OK-Seq RFD of TRIs still had increased amplitude and slope compared to transcription activity-matched cTSSs, similar to total cTSSs (Supplementary Figure S4D). Thus, transcriptional activity alone does not explain the observed origin enrichment and early replication timing observed at TRITSSs.

#### TRIs are located near early replication origins

OK-Seq and TimEX define both TRIs and cTSSs as origin-rich and early replicating. To confirm these results, we assessed DNA replication using EdU-Seq data from mouse primary B cells (98). EdU-Seq assesses early S phase origin firing by stimulating G0 splenic B cells to enter S phase in the presence of hydroxyurea (HU) to slow replication and the thymidine analog 5-ethynyl-2’-deoxyuridine (EdU) to label nascent DNA (100); hence, a high EdU signal indicates early replication. We found that EdU-Seq signal in HU-treated cells is highest upstream of the TSSs with strong depletion of signal at the center and an increase in signal after the TSSs (Figure 3E). The pattern at TRIs and TRITSSs was more pronounced than cTSSs. These results suggest that TRIs initiate replication earlier than cTSSs but take longer to complete replication downstream; this interpretation is supported by TimEX analysis (Figure 3C, D). We observed similar patterns using EdC-Seq—a variant of EdU-Seq using a cytosine analogue for labeling—from HU-treated cells, indicating that this pattern is independent of sequence (Supplementary Figure S4E) (98). We also observed a dip EdU-Seq signal at TRIs and cTSSs in the absence of HU (Supplementary Figure S4F). From this data, we propose that TRIs experience frequent replication fork stalling with or without exogenous stress.

#### TRIs exhibit high levels of replication protein A in cells experiencing replication stress

Stalled replication forks accumulate single-stranded DNA (ssDNA) that can be bound by replication protein A (RPA) which then acts as a signal for other processes to repair the stalled replication forks (101). Mapping RPA binding to genome-wide by anti-RPA32/RPA2 ChIP-Seq has been used to define where ssDNA is formed in primary B cells in response to DSB formation or HU-induced replication stress (60,67,102,103). In HU-treated cells, TRIs and TRITSSs exhibit significantly higher levels of RPA signal than cTSSs with the signal centered at the TSSs (*P* ≤ 1.0 × 10^–250^, Wilcoxon rank sum test; Figure 3F). In yeast, RPA accumulates on the lagging strand during HU-induced replication stress (104). In contrast, RPA from untreated cells from a separate dataset showed a depletion of RPA at TRIs and cTSS, with RPA signal significantly lower at TRIs (Supplemental Figure S4G). To determine if RPA association shows a similar strand bias in mammalian cells, we next measured RPA signal along plus and minus strand surrounding TRIs. We observed that the RPA signal peak was upstream of the TRITSS center for plus strand genes, and downstream of the TRITSS center on minus strand genes when treated with HU (Supplementary Figure S4H). These results are consistent with RPA accumulation on the lagging strand as observed in studies of stalled replication forks in yeast (104,105).

### DNA double-strand break formation at TRIs

#### Spontaneous DSBs are enriched at TRIs

**Figure 4.**
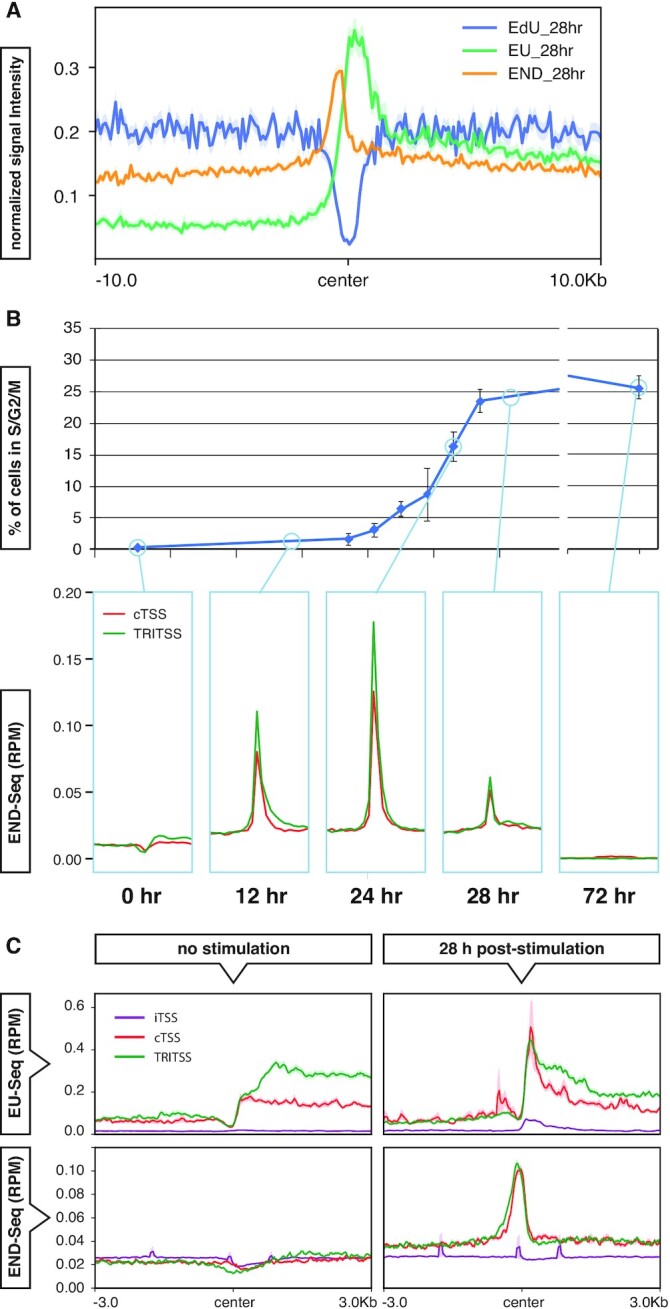
DNA breaks at TRIs. (**A**) Median signal profiles centered at TRIs of EdU-Seq (blue), EU-Seq (green) and END-Seq (orange) from 28 h NT cells. The EdU-Seq and END-Seq signal was multipled by 8 and 4 respectively so the signals can be seen clearly on the same scale. (**B**) Top graph showing the percentage of B cells in S and G2/M phases measured by PI incorporation and analyzed by FACS. Bottom of the panel showing END-Seq signal profile at 0, 12, 24, 28 and 72 h time points from NT cells centered near TSSs of TRITSSs (green) and cTSSs (red). (**C**) Median signal profiles of EU-Seq (top) and END-Seq 0 mM HU (bottom) of non-replicating resting (left) and 28 h activated replicating mouse B cells (right) centered near TSSs of TRITSSs (green), cTSSs (red) and inactive TSSs (purple). *All profile plot analyses are performed in mBCs. Shaded areas on the unfilled line plots are the standard error.

TRIs are putative areas of transcription–replication conflicts and potential sites of stalled replication forks and DNA DSBs (106). To assess if TRIs are enriched for DSBs, we analyzed published END-Seq datasets which map exposed DNA ends genome-wide in murine B cells (57). Over 87% of TRIs overlapped with END-Seq signal peaks, a 33-fold enrichment over random chance (Supplementary Figure S5A). To define where DSBs occur relative to transcription and replication at TRIs, we compared END-Seq NT, EU-Seq NT and EdU-Seq NT datasets from non-treated cells harvested 28 h post-stimulation. Signal intensities were adjusted to fit all three sample types on the same scale. We found that the EdU-Seq signal decreased as it approaches TRIs, while EU-Seq signal sharply increased at the center of TRIs (Figure 4A). END-seq signal was highest slightly upstream of where replication and transcription coincide at TRIs. From this data, we conclude that TRIs are enriched for spontaneous DSBs immediately upstream of where transcription begins and replication timing delays occur.

#### Spontaneous DSBs at TRIs and cTSSs correlate with transcription and replication activity

We next compared the END-Seq signal at TRIs and cTSSs with entry into S phase to determine if DSB accumulation correlates with DNA replication. In response to antigen stimulation, naïve splenic B cells resting in G0 first undergo an increase in transcription, then enter the cell cycle and replicate (107). Flow cytometric analysis of DNA content shows that DNA replication initiates around 14–16 h post-stimulation, with ∼16% of cells in S phase by 24 h (Figure 4B, top panel, Supplementary Figure S5B). END-Seq signal positively correlated with cells entering S phase at 12 and 24 h post-stimulation for both TRIs and cTSSs but the END-Seq pattern is not visible at 72 h when cells are asynchronously growing (Figure 4B, lower panels) (57,98,108). These results indicate these DSBs are replication-dependent and do not simply represent transcription-induced damage.

To determine if both replication and transcription are required for DSB formation, we compared END-Seq and EU-Seq data from replicating and non-replicating cells at TRITSSs, cTSSs and inactive TSSs (iTSSs) (57,98). We found EU-Seq signal at TRIs and cTSSs in non-replicating cells (Figure 4C, upper left) but no enrichment of END-Seq signal (Figure 4C, lower left). In replicating cells, we found similar levels of END-Seq signal accumulation at actively-transcribing TRIs and cTSSs, but not iTSSs which had minimal transcription by EU-Seq (Figure 4C, right panels). This is distinct from END-Seq data at 12 and 24 h where we observe higher END-Seq signal at TRIs than cTSSs. This difference appears to be transient, therefore we conclude break levels are largely similar between TRIs and cTSSs. We also observed comparable levels of END-Seq signal at TRITSSs and cTSSs from cells exposed to HU (Supplementary Figure S5C). These results suggest that DSBs formed at TRIs and cTSSs accumulate only when replication and transcription are both active. The Ataxia telangiectasia and Rad3-related checkpoint kinase (ATR) plays a critical role in the repair and/or restart of stalled replication forks (60,109). To determine if loss of ATR enhanced DNA damage at TRIs, we analyzed END-Seq data at TRIs and cTSSs in the absence and presence of the ATR inhibitor (ATRi) AZ20. We observed an increase in the END-Seq signal in cells treated with 10 μM ATRi at both TRIs and cTSSs, consistent with the notion that ATR helps suppress DNA damage arising at these loci (Supplementary Figure S5D) (98).

To investigate how DSBs are distributed around TRIs, we analyzed 24 hr END-Seq signal on the positive and negative DNA strands. We found that the END-Seq signal at TRIs was somewhat higher than cTSSs and had a wider distribution than cTSSs, particularly on the Crick strand (Supplementary Figure S5E). Together, these results suggest that DNA breakpoints are more localized at cTSSs. In a previous study, END-Seq signal accumulated unevenly around poly(dA:dT) tracts in response to HU with a ratio of ∼2:1, suggestive of fork collapse from a stalled DNA polymerase (98). However, the distribution of END-Seq signal at TRIs was 1:1; therefore, we speculate that polymerase stalling is not the dominant cause of TRI-associated DSBs.

#### DSBs form independently of TOP2

Topoisomerase II (TOP2) relieves topological stress through its DNA cleavage-religation activity; inhibition of TOP2 re-ligation with etoposide leads to single-strand breaks (SSBs) and DSBs (110). To assess if TOP2 activity contributes to DSB formation at TRIs, we analyzed END-Seq datasets for etoposide-treated WT and TOP2B knockout (TOP2BKO) mouse B cells as a function of time post B cell simulation (57). When non-replicating WT cells were exposed to etoposide, END-Seq signal increased in regions flanking the TSS but not at the start site itself, suggesting these DSBs are associated with transcription only (Supplementary figure S4C). When replicating WT cells were treated with etoposide, the END-Seq signal again accumulated in the regions flanking TRI/TRITSSs with no noticeable increase in the center; however, in TOP2BKO cells the END-Seq signal was still centered at the TSS (Supplementary Figure S5F) (111). These results suggest that the flanking DSBs forming around TRIs are associated with transcription and TOP2B activity, while DSBs directly at the center of TSSs are largely TOP2B-independent.

#### TRIs are enriched for early replicating fragile sites

It is hypothesized that chromosomal fragile sites—genomic regions experiencing recurrent DNA breaks due to replication stress—may be a result of transcription–replication collisions (112). We analyzed the association of TRIs with 615 HU-sensitive putative early-replicating fragile sites (ERFSs) that overlap a total of 126 624 kb (60) and 17 known aphidicolin-sensitive late-replicating common fragile sites (CFSs) overlapping 167 578 kb in total (61–66). For this study we considered only CFSs validated by FISH in at least one study, as no genome-wide analysis of aphidicolin-induced common fragile sites has been undertaken in mouse. Fragile sites are very large genomic regions ranging from 50 kb to over 2 Mb while the median TRI length was 1180 bp, therefore we investigated the overlap in basepairs instead of the number of fragile sites and TRIs. We found a 2.8-fold enrichment of TRIs in ERFS-associated regions, but no enrichment of TRIs in CFS regions (Supplementary Figure S6A, B). Of note, TRIs overlap 3/7 ERFSs validated as hypersensitive to exogenous replication stress by fluorescent in situ hybridization—BCL2, BACH2 and IKZF1 (60,113). The enrichment of TRIs for ERFSs but not CFSs is consistent with OK-Seq and TimEX analyses indicating TRIs are origin-rich and early replicating. ERFSs are enriched for gene pairs exhibiting divergent transcription visible as two TSSs within 3 kb of each other (60). Similar to ERFSs, we found TRITSSs had divergently-transcribing gene pairs, such as *Thap4/Atg4b* (Figure 2C). To determine if TRIs overlapping divergently transcribed genes had elevated signals of DNA damage or replication stress, we next compared RPA and END-Seq signal between TRIs overlapping a single annotated TSS, TRIs overlapping two convergent TSSs, and TRIs overlapping two divergent TSSs. We found RPA and END-Seq signal were similar for all three classes (Supplementary figure S6C, D). These results indicate that TRIs overlapping divergent and convergent gene pairs experience similar levels of fork stalling and breakage as TRIs with a single TSS.

### Sequences and mutations at TRIs

#### GC sequences are enriched at TRIs

**Figure 5.**
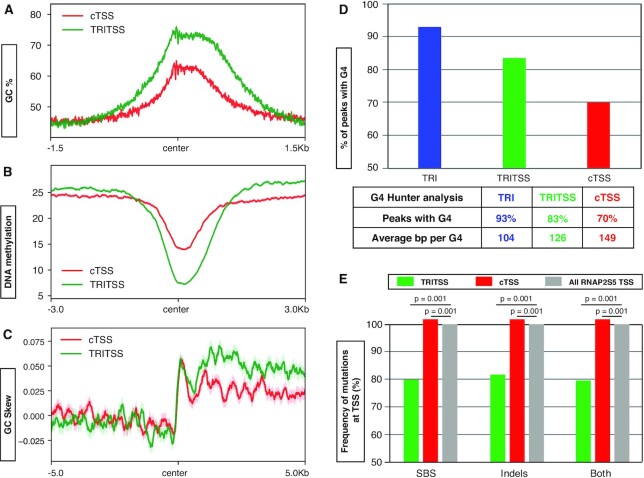
Sequences and mutations at TRIs. (**A**) Median signal profile of percent GC of the mm10 mouse genome centered near the TSSs of TRITSSs (green) and cTSSs (red). (**B**) Median DNA methylation signal profile of mouse B cells centered near the TSSs of TRITSSs (green) and cTSSs (red). For TRIs versus cTSS, *P* < 1.0 × 10^–250^ and TRITSS versus cTSS, *P* = 8.3 × 10^–238^; Wilcoxon rank sum test. (**C**) Median signal profile of GC skew of the mm10 mouse genome centered near the TSSs of TRITSSs (green) and cTSSs (red). GC skew of TRIs and TRITSS are significantly higher than cTSS (*P* = 1.17 × 10^–66^ and *P* = 1.48 × 10^–67^ respectively, Wilcoxon rank sum test). (**D**) Table and bar graph of the predicted frequency of G-quadruplex formation at TRIs, TRITSSs and cTSSs. (**E**) Bar graph of single basepair substitutions (SBS) and short insertions/deletions (indels) at TRIs, TRITSSs and cTSSs normalized to cTSSs showing empirical *P*-value. *Shaded areas on all unfilled line plots are the standard error.

Repetitive DNA elements such as trinucleotide repeats and long inverted repeats can form secondary structures capable of inducing replication fork blockage (114). We examined the nucleotide content and found that TRITSSs are significantly more GC-rich than cTSSs, with enrichment peaking at TRI centers (*P* < 1.0 × 10^–250^, Wilcoxon rank sum; Figure 5A; Supplementary Figure S7A). Additionally, 99% of TRIs overlap with CpG islands, a 78-fold enrichment over random chance (Supplementary Figure S7B). CpG islands correlate with low DNA methylation levels (115). Consistent with this notion, TRITSSs had significantly lower DNA methylation than cTSSs (116) (*P* = 8.3 × 10^–238^, Wilcoxon rank sum test; Figure 5B). We next measured GC-skew, an asymmetrical distribution of nucleotides where guanines are more abundant than cytosines. GC-skew has been associated with R-loops, CpG island promoters and prokaryotic replication origins (78,117). We found a sharp increase to a positive GC-skew at the center of both TRITSSs and cTSSs (Figure 5C). This enrichment is not suprising as GC skew is associated with both coding regions and replication origins in mammals (118–121). However, TRITSSs exhibited significantly higher GC-skew than cTSSs within gene bodies (*P* = 1.48 × 10^–67^, Wilcoxon rank sum test; Figure 5C). G-quadruplex (G4) motifs can form secondary structures when guanine-rich sequences form a helical shape stabilized by G-G base pairing and have been implicated in genome instability (122). We used G4 Hunter to predict G4 structure formation, and found that 93% of TRIs and 70% of cTSSs can potentially form G4s (77). TRIs average 1 possible G4 per 104 bp, while cTSSs could form 1 possible G4 per 149 bp (Figure 5D). Using Homer, we also found an enrichment of two similar GC-rich sequences, CCGCCGCC and GGCGGCGG, in both TRIs and cTSSs but the frequency was higher in TRIs. (Supplementary Table S3). These results show that TRIs are enriched for GC content, potential secondary structure-forming G4 sequences, and GGC trinucleotide repeats.

#### TRIs accumulate deletion mutations

TRIs and cTSS are enriched for DNA breaks by END-Seq; therefore, it is possible they accumulate mutations. We analyzed sequence variants containing single basepair substitutions (SBSs) and short insertions/deletions (indels) in four independent datasets from mouse primary B cells in 1 kb windows—two TimEX sequencing results and two datasets used as input controls for ChIP-Seq experiments (73,98). Compared to all RNAP2S5-associated TSSs, TRITSSs harbored fewer SBSs and indels (*P* < 0.001, Figure 5E, empirical *P*-value). However both mutation types were increased at cTSSs (*P* < 0.001, empirical *P*-value; Figure 5E). Upon further analysis of indels, we found an enrichment of deletions (Supplementary Figure S7C, Supplementary Table S4). Together, these results indicate that TRITSSs accumulate fewer mutations than cTSSs, but TRI gene bodies accumulate more mutations than cTSS genes.

The Catalogue Of Somatic Mutations In Cancer (COSMIC) has developed a set of bioinformatic tools to perform variant analysis and identify specific mutational signatures from next-generation sequencing data (123). To investigate the specific types of mutations occurring at TRIs and cTSSs, we analyzed the SBSs and indels using the COSMIC SigProfiler software suite. We found an over-representation of C(C > A)G mutations in TRITSS regions and full genes compared to cTSSs (Figure S7D—blue bars, Supplementary Table S4). TRITSSs also had more 3 bp deletions at repeats than cTSSs (Supplementary Figure S7E—pink/orange lines, Supplementary Table S4). These distinct mutations may occur because TRIs have more trinucleotide CCG repeats therefore they are overrepresented, or because CCG/GGC motifs can cause DNA polymerase stalling (124). Thus, though mutations are less frequent in TRITSSs, they preferentially accumulate small deletions in repetitive DNA sequences.

#### TRI genes overlap with cancer drivers

We next probed a dataset from the Mouse Tumor Biology Database (MTBD) that listed 23 625 mutations (6525 unique gene names) from sequenced mouse tumors (71). We found that 57.3% of TRI genes harbor mutations, a 3.4-fold enrichment over random chance (Supplementary Figure S7F). Insertions were overwhelmingly the most abundant mutation in the MTBD dataset at 73% of all mutations (Supplementary Table S5). When analyzing MTBD mutations at TRIs we observed an underrepresentation of insertions (79%) and an overrepresentation of deletions (227%), nonsense mutations (356%) and point mutations (214%) (Supplementary Table S5, Supplementary Figure S7G). Since TRI gene mutations associate with mouse tumors, we next explored the Sleeping Beauty Cancer Driver DataBase (SBCDDB) which identified 1231 cancer drivers (72). Here we observed a 29% overlap with SBCDDB cancer drivers, a 9.2-fold enrichment over random genes. These results show TRI regions accumulate specific mutation subtypes in murine tumors.

#### TRI gene set enrichment analysis

DNA damage and accumulation of somatic mutations have been hypothesized to be involved with cancer (125). Using MouseMine, we found that TRI genes are highly enriched for preweaning lethality, abnormal survival, mortality and aging phenotypes in mice (Supplementary Table S6) (80). We found that TRI genes were enriched in KEGG pathways associated with cancer and transcription factor protein-protein interactions (PPIs) with cancer associated genes such as *Tp53*, *Brca1* and *Myc* (Supplementary Table S7). Our results also showed an association of TRI genes with stem cell pluripotency factors; pluripotency factors can be induced in cancers and associate with poor treatment outcomes (Supplementary Table S8) (126). This association of TRIs with cancer is supported by the overlap between MTB tumor mutations and SBCDDB cancer drivers. Chromatin structure, post-translational modifications and gene expression have been implicated in aging and cancer. We also observed an enrichment of TRI genes in these processes as well as PPIs with *Ep300* and *Hdac2* (Supplementary Table S9) (127).

## DISCUSSION

In this study, we developed a method to identify genomic locations where transcription and replication machinery colocalize genome-wide, identifying 1,009 independent TRIs in primary mouse B cells. TRIPn-Seq can be applied to any proliferating cell type, as it relies on incorporation of modified nucleotides into nascent replication. A subset of TRIs overlap two unique annotated genes, therefore we identified TRIs at 1198 active genes. However, these results do not rule out transcription–replication problems at the other 12 957 active genes. Rather, TRIs may represent areas of prolonged or complex interactions. Indeed, the bidirectional transcription so prevalent at TRIs strongly increases the chance of conflicts with replication machinery in proliferating cells.

TRIs harbor distinct patterns of chromatin features which are distinct from other transcribed genes. In particular, TRIs are characterized by a bimodal pattern for RNAP2s5, R-loops by DRIP-Seq, P300, GCN5, HDAC1 and HDAC2. Transcriptional activity influences the placement of proteins involved in chromatin architecture; we see a similar bimodal pattern in RAD21 and CTCF at TRIs. From the RNAP2s5 ChIP-Seq libraries generated here we cannot confirm the simultaneous binding or direction of two RNAP2s5 molecules on the same DNA strand, but the presence of two RNAP2s5 populations is supported by the bidirectional overlapping transcription measured by EU-Seq and GRO-Seq (Figure 2B, C and Supplementary Figure S2B, C). This transcriptional landscape indicates that multiple RNA polymerase complexes are engaged on the template and non-template strand surrounding TRITSSs. Multiple RNAPs moving in a codirectional or convergent orientation with respect to replication are more difficult impediments to replication than a single RNAP, and may explain why TRIs were detected at these locations (16). Divergent transcription has also been shown to increase genome instability and interactions with replication machinery likely exacerbates this (128). Although transcription and replication machineries may interact at all transcribed loci, it is possible that only a specific orientation of RNAP2s5 causes prolonged or increased frequency of fork stalling at TRIs. Overall, TRI genes show higher levels of nascent transcription relative to cTSS genes, as well as chromatin marks associated with active transcription (Figure 2B, Supplementary Figure S3). Thus, higher levels of RNAP sense transcription may increase interactions with ongoing replication, increasing TRI frequency.

TRIs and TRITSSs also exhibited a bimodal pattern for DNA:RNA hybrid formation more similar to RNAP2s5 ChIP-Seq signal than active transcription as measured by EU-Seq or GRO-Seq where the upstream signal is low. GC skew has been associated with R-loop formation, yet we only observed GC skew downstream of TRITSSs (117). This begs the question, what stimulates R loop formation upstream of TRITSSs? One possibility is that upstream R loops are stimulated by RNAP2s5 pausing. Paused RNAP2 could anchor nascent RNAs in place, promoting interaction with the template strand. Recent studies also suggest that DNA supercoiling can drive RNA:DNA hybrid formation in sequences without significant GC skew; this may relieve torsional stress by allowing the DNA strand to twist around the RNA (129). Thus, regions upstream of TRITSSs forming R-loops may ‘absorb’ negative supercoiling, while GC skew promotes R-loop formation downstream of the TRITSS. We hypothesize that R-loops at TRIs may be a consequence rather than a cause of transcription–replication interactions. Both transcription and replication generate negative supercoiling behind elongating complexes and positive supercoiling in front suggesting a reliance on topoisomerases to maintain genomic integrity. Intriguingly, analysis of END-Seq data from etoposide-treated TOP2BKO cells show that DSBs still accumulate at the TSSs, even though flanking DSBs decrease from WT (Supplementary Figure S5C) (130,131). This evidence may imply that TOP2B is not the sole enzyme creating DSBs at TRIs; in the absence of TOP2B, additional enzymes may process topological stress at these sites such as TOP1 as shown in recent studies (132).

Genetic and epigenetic signatures of TRIs point to a chromatin landscape providing multiple roadblocks to efficient replication, leading to fork stalling and collapse. This is reflected in OK-Seq analyses which show a distinct pattern of replication around TRIs with origins enriched upstream of the TSSs, and a strong increase in termination throughout gene bodies and TTSs (Figure 3A). This pattern is consistent with prior publications showing replication termination is enriched within gene bodies in response to HU-induced replication stress (97). Regions upstream of TRIs appear to be some of the earliest replicating loci in the genome as measured by EdU-Seq, EdC-Seq and TimEX experiments. Early replicating regions are associated with high transcription, increasing the chance of transcription–replication conflicts in early S phase (133). All replication datasets examined show subtle but consistent dips in replication efficiency at the center of TRIs (Figure 3B-E, Supplementary Figure S4A–D). Similar studies measuring DNA replication and transcription throughout S phase in human fibroblasts also show a strong delay in replication around TSSs (134). Thus, we hypothesize TRIs are difficult-to-replicate regions enriched for RF stalling. Indeed, TRIs accumulate extensive RPA—a hallmark of replication fork stalling (135).

Consistent with these observations, TRIs show extensive overlap with ERFSs, potential early replicating fragile sites mapped by DNA repair protein association in response to acute HU treatment (60). In contrast, we found no overlap of TRIs with late-replicating CFSs. This is expected in light of recent reports indicating that CFS breakage is strongly influenced by alterations in replication timing and origin firing over these regions in the presence of aphidicolin. Instead of replication-transcription collisions inducing DSB formation by replication fork collapse, cells enter mitosis without completing replication at these regions (29–31). ERFSs and TRIs also share genetic and epigenetic similartiies. Similar to ERFS, TRIs are enriched for histone marks of active transcription and open chromatin, exhibit hgher GC content and are enriched for CpG islands.

**Figure 6.**
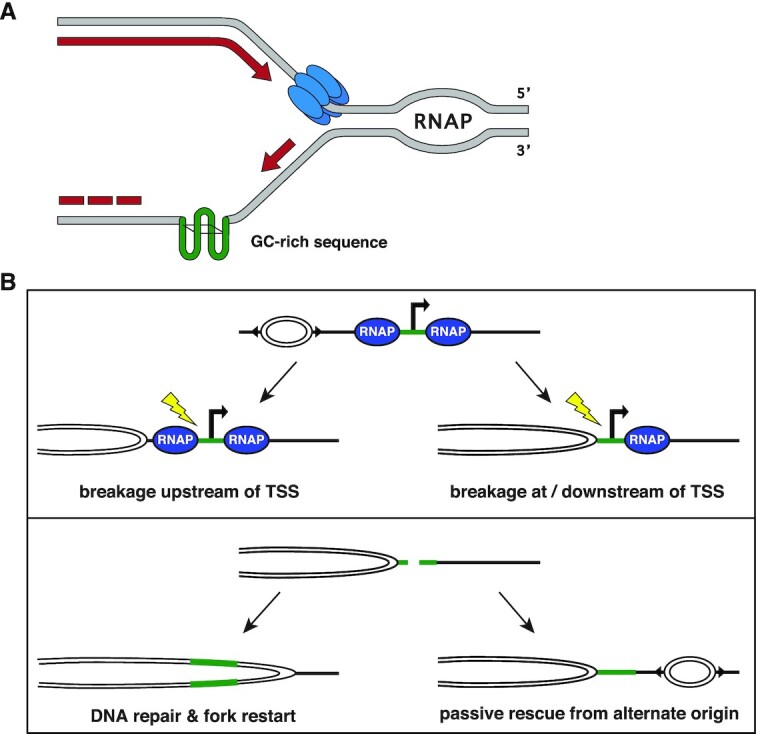
Summary of features at TRIs and model of TRIs. (**A**) Graphic summary of chromatin features at TRIs and chromatin features of cTSSs. (**B**) Graphic representation of a TRI where the replicative helicase is stalled by an RNAP molecule and the DNA polymerase is stalled by a complex GC-rich sequence or structure (green) simultaneously.

We propose that TRIs represent genomic loci which can stall both the replicative helicase and DNA polymerase. In our model, replication initiates upstream of active genes. Near the TSS of TRIs, the helicase of a moving replisome may stall on the leading strand, while the lagging strand DNA polymerase can stall at a complex GC sequence or DNA:RNA hybrid (Figure 6A). This is consistent with recent reports showing G-quadruplex formation on the non-template strand can promote R-loop formation and vice-versa (136). Alternatively, increased ssDNA formation by replication stalling at TRIs may promote the formation of G-quadruplexes and/or R-loops at TRI loci (137–139). Future studies temporally separating replication and secondary structure formation will help elucidate if fork progression or stalling promotes their formation. After prolonged fork stalling, a double strand break can form and the RNAP2s5, replication fork, or both are displaced. There is a short amount of resection (<500 bp), RPA is loaded onto the ssDNA, and the damage is repaired by microhomology-mediated end joining (MMEJ). How is replication at TRIs completed if they are such roadblocks to fork progression? Replication across TRIs can be completed by fork restart if the replisome remains associated, or by passive replication from adjacent origins (Figure 6B). Another possibility is that TRIs are bypassed and replicated later through a non-conventional replication mechanism, generating a single stranded gap intermediate. TRIs and cTSSs exhibit similar levels of DSBs by END-Seq (Figure 4C). Taken together with the increase in RPA signal, these results suggest TRIs are often sites of prolonged fork pausing that only occasionally results in DSB formation during unperturbed conditions or HU-induced stress. In agreement with our findings, recent evidence indicates that a subset of TSSs are replicated in G2/M (134). EdU-Seq also provides some evidence for this possibility as downstream signal is higher than at the center (Figure 3C). Alternatively, downstream replication forks may replicate this area resulting in fork termination at TRIs (Figure 6B).

DSBs need to be repaired to preserve genome integrity and TRIs show higher flanking signals of H4K16Ac, H3K36me2, H3K79me2 and H4K20me1 all of which are involved in non-homologous end joining (NHEJ) during DSB repair when compared to cTSSs (Supplementary Figure S3) (94). However H3K79me2 and H4K20me1 also correlate with faster transcriptional elongation and longer gene length, the latter also being a feature of TRIs (95,96)(Supplementary Figure S1D). TRIs also have considerably more RPA in HU treatment than cTSSs indicating persistent ssDNA formation. This accumulation of RPA-bound ssDNA may be a hallmark of DSB repair by homologous recombination (HR) at TRIs or simply an accumulation of stalled replication forks (101). Alternatively, RPA is also present at sites of alternative end joining involving ssDNA tails which contributes to deletions and translocations (140).

TRIs are enriched for small deletions, consistent with the increase in END-Seq signal and NHEJ-associated chromatin marks. TRIs have a wider distribution of DSBs compared to cTSS which may imply that TRI-associated DSBs are generated by alternate means, or the DSB ends are processed more extensively for repair. Processing of DSB ends revealing ssDNA would make TRI breaks substrates for MMEJ, a mutagenic form of NHEJ with a propensity for inducing microdeletions (141). Indeed, MMEJ repairs breaks at collapsed replication forks, and convergent transcription–replication interactions increase the frequency of deletion events (142,143). The enrichment of trinucleotide repeats at TRIs are also likely to play a role in the increase in deletions, as MMEJ requires short regions of homology. Replication slippage also occurs at trinucleotide repeats, resulting in small deletions as well as expansions of repeated sequences and requires disruption of continuous replication. Our mutational signature analysis shows that there are patterns that match COSMIC samples experiencing replication slippage (Supplementary Figure S7E) (124,144). It is therefore surprising that we find fewer single nucleotide variants at TRIs than cTSS, since they harbor characteristics of MMEJ (145). Though TRIs have elevated DSBs by END-Seq and high levels of RPA, TRI genes are depleted for single basepair mutations and indels. One possibility is that TRIs experience less mutagenic DNA repair. TRI genes are highly enriched for preweaning lethality, abnormal survival, mortality and aging phenotypes. Thus mutations in TRI genes may induce cell lethality, leading to fewer mutations in sequencing analysis. Further experiments assessing DSB repair pathway choice at TRIs will define the contribution of NHEJ, HR and alternative repair pathways at these sites.

TRIs may also have a role in promoting genome integrity. Codirectional transcription–replication collisions can induce replication fork restart and activate the DNA damage response (27,146). Thus, TRI-induced fork pausing at TSSs may minimize co-directional collisions from occurring within the coding regions of essential genes. This is similar to replication fork barriers that are found in active ribosomal DNA termination regions which prevent convergent TRIs within the gene (147). If the cell survives, acquired mutations can persist and potentially accumulate leading to aging phenotypes. Changes near TSSs are more likely to induce expression changes which could eventually reach a critical limit resulting in gene silencing or overexpression. At TRI genes such as *Cop1*, a negative regulator of tumor suppressor gene *Tp53*, this could disrupt the balance and drive tumor formation. Mechanistic studies investigating TRI-associated DSB formation and repair will help untangle how transcription and replication influence genome instability, tumorigenesis and aging.

## DATA AVAILABILITY

TRIPn-Seq, DRIP-Seq and ChIP-Seq data has been deposited to the Gene Expression Omnibus (GEO) database under the accession GSE161410 and the Flow Repository under ID# FR-FCM-Z3GS. Previously published data analyzed in these experiments are from GSE100262 (116), GSE116321 (98), GSE129524 (111), GSE99197 (57), GSE82144 (73), SRA: PRJNA326246 (108) and mm10 GC percent and CpG Island annotations (59).

## Supplementary Material

gkac035_Supplemental_Files
