# Supplementary material for: Genomic patterns of transcription–replication interactions in mouse primary B cells
Source: Nucleic Acids Res. 2022 Jan 31;50(4):2051–73. doi: 10.1093/nar/gkac035 (PMC8887484; doi:10.1093/nar/gkac035)
Supplement: gkac035_Supplemental_Files [file gkac035_supplemental_files.zip › Supplementary figures_St Germain.pdf]

Supplementary Figure 1

A

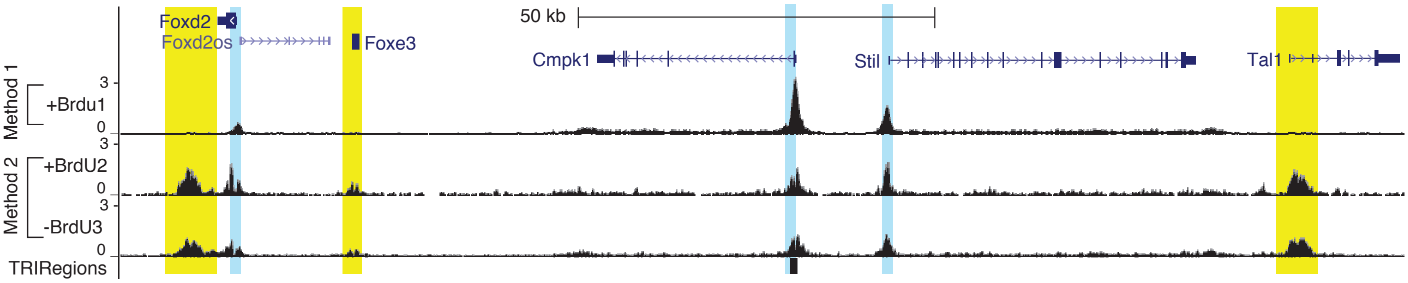

B

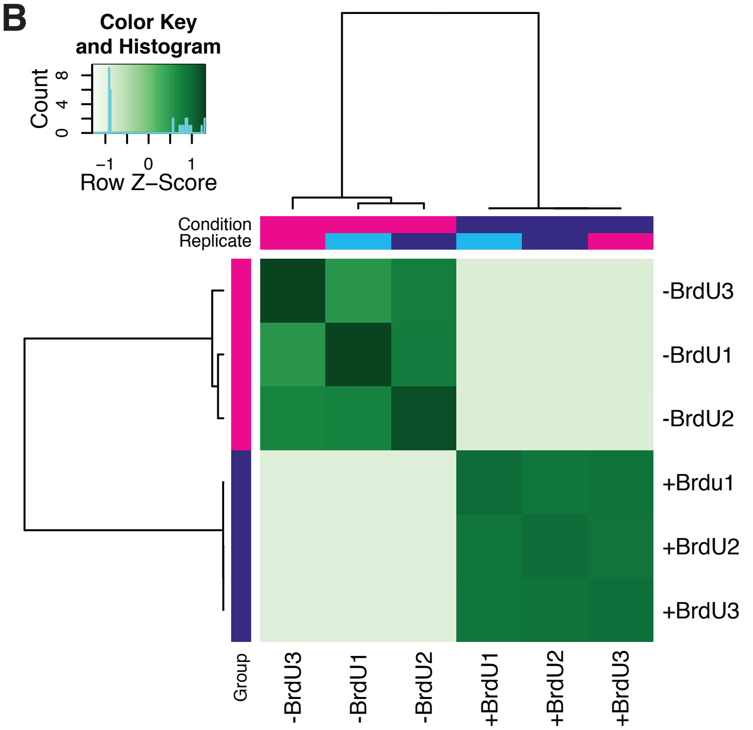

C

TRI Properties	
# of input peaks	76,154
# with FDR < 0.05	357
# overlapping TSS	334
Median (bp)	528
Mean (bp)	588
Min (bp)	200
Max (bp)	1,676

D

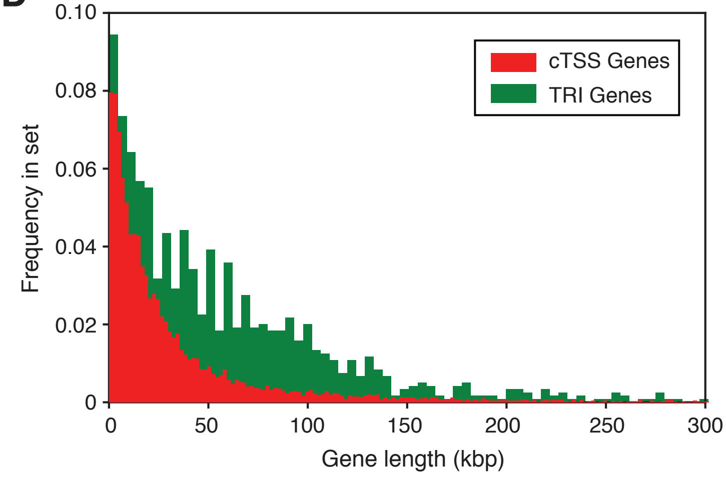

E

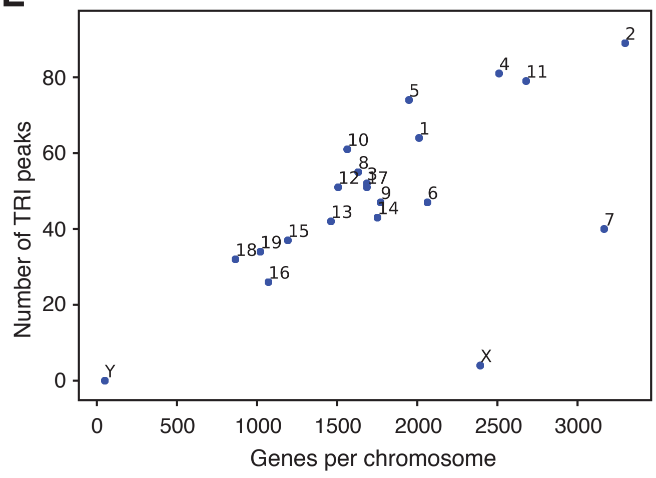

Supplementary Figure 2

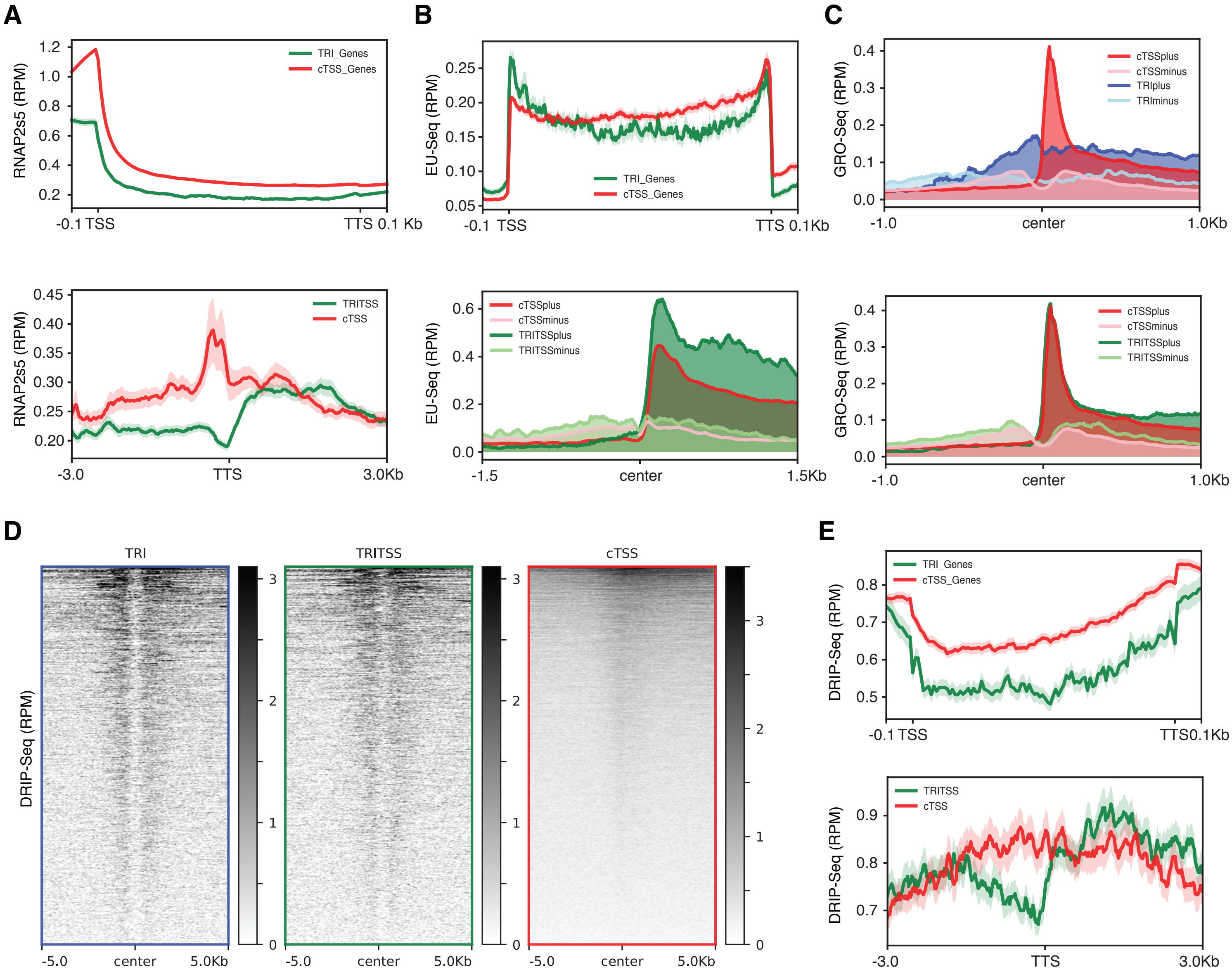

Supplementary figure 3

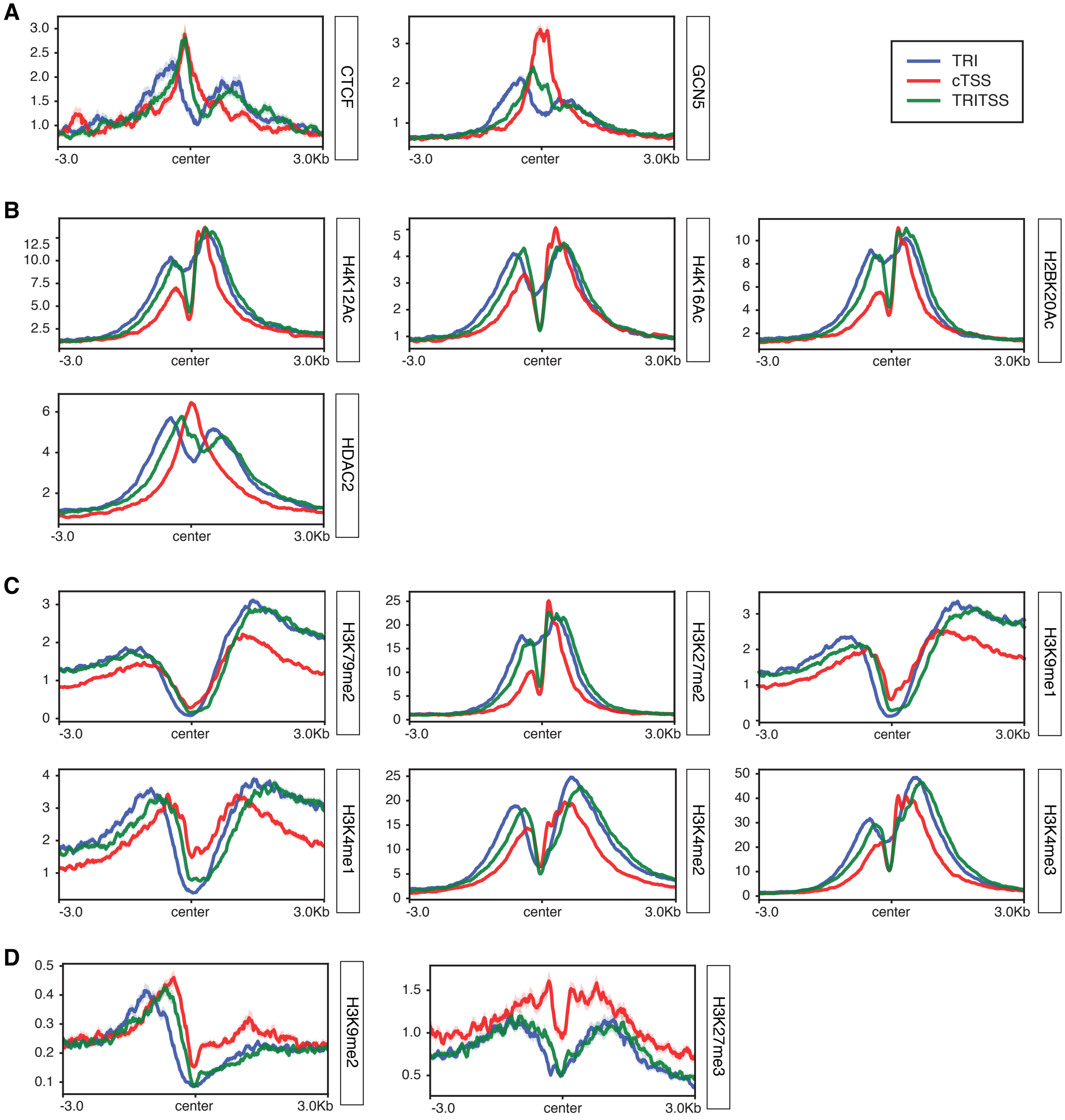

Supplementary Figure 4

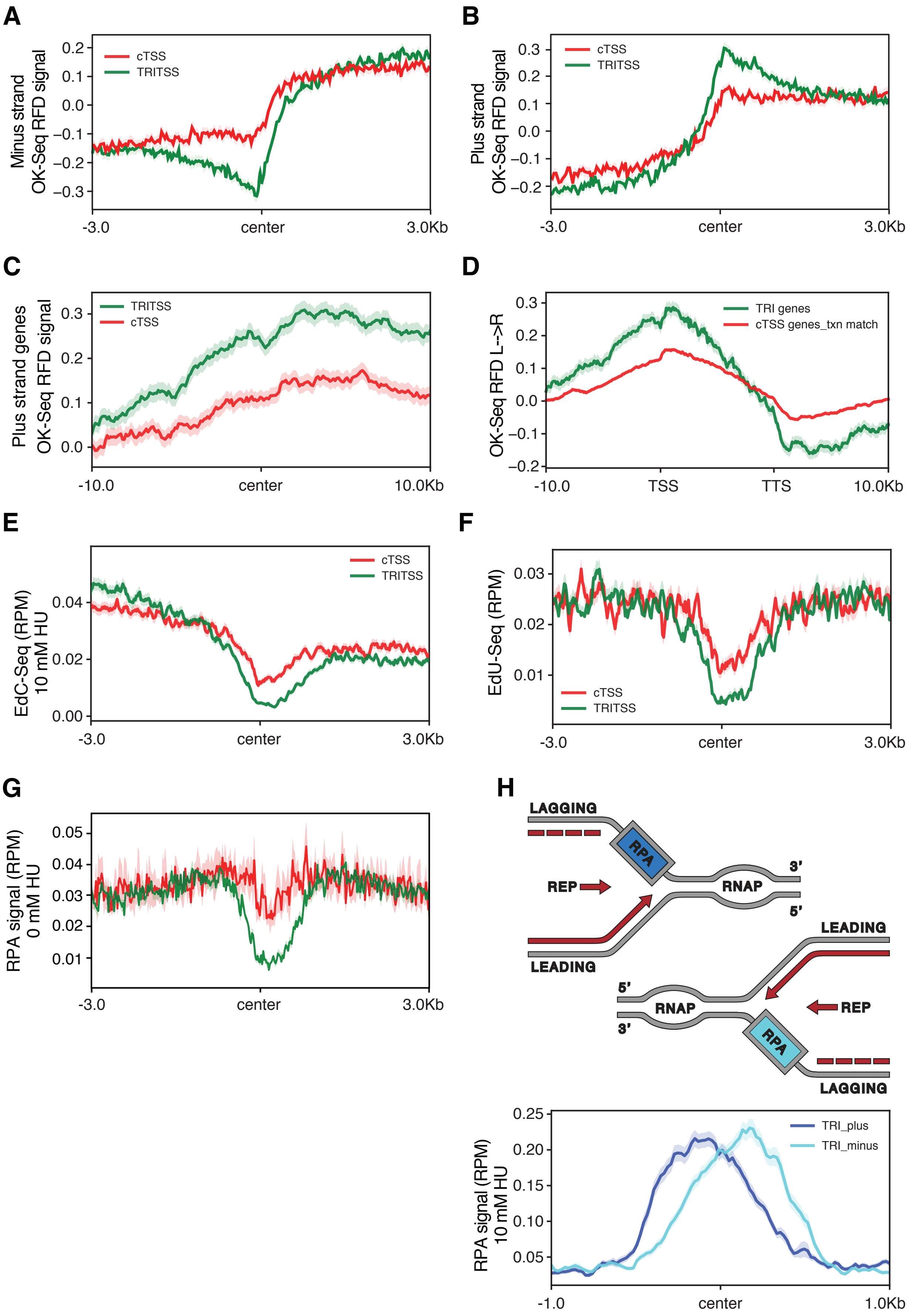

Supplementary Figure 5

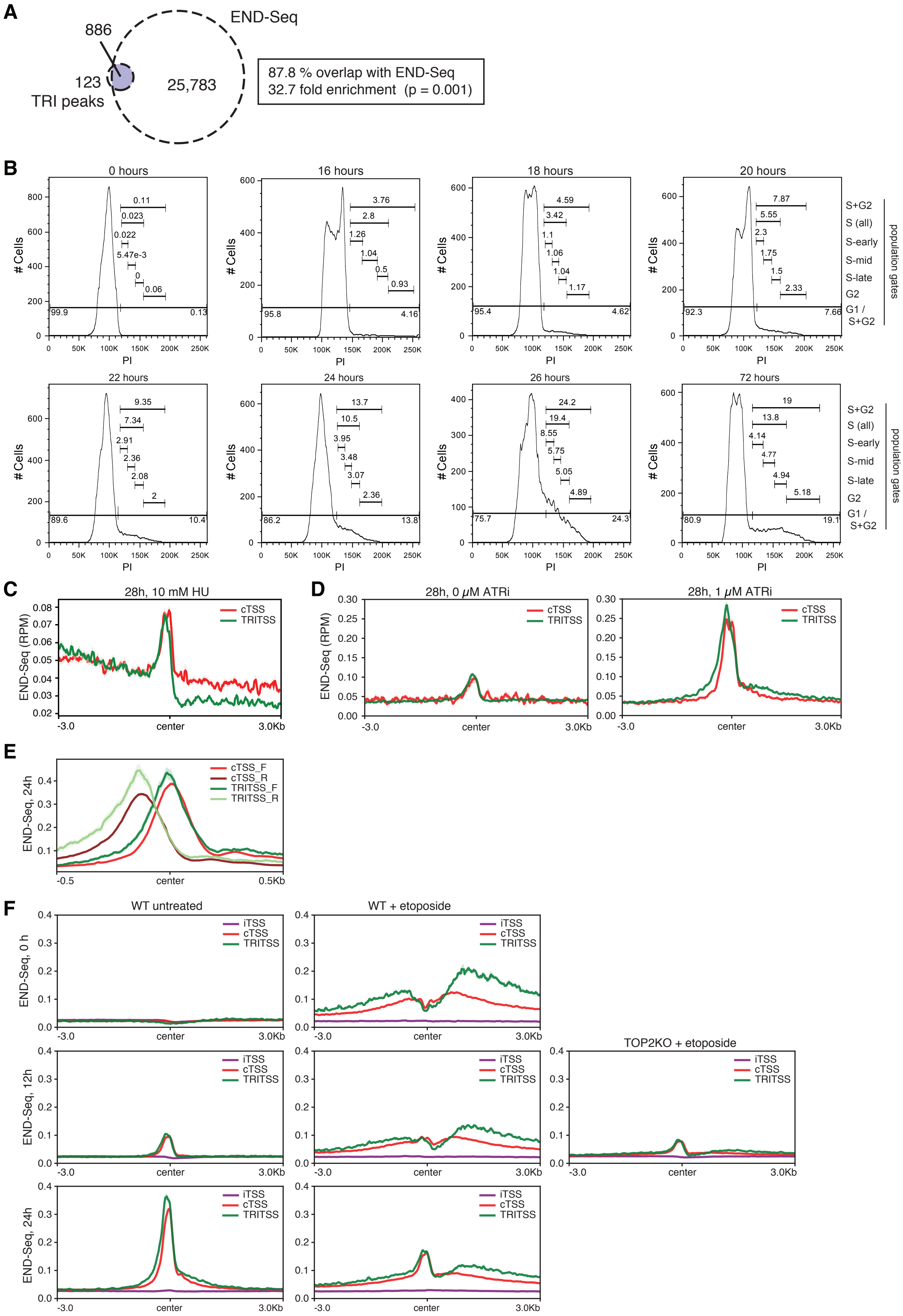

Supplementary Figure 6

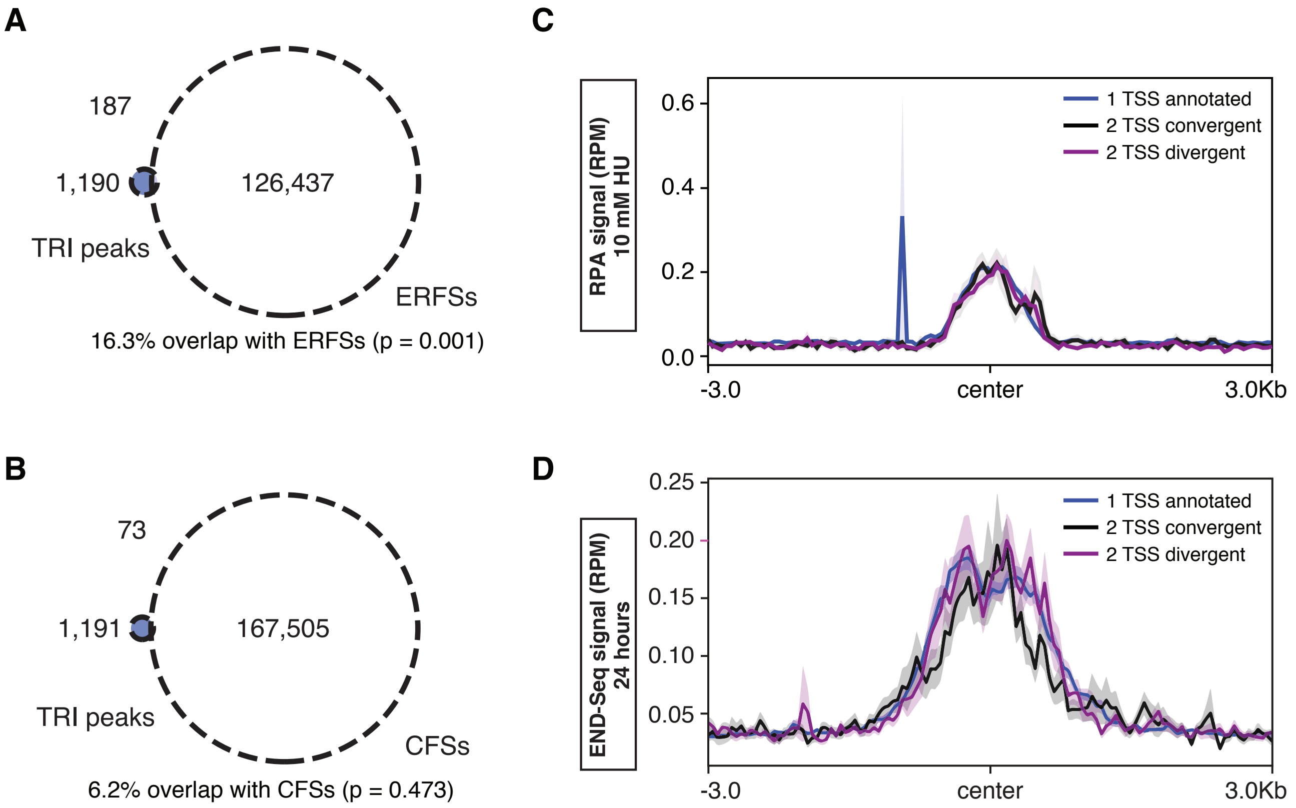

Supplementary Figure 7

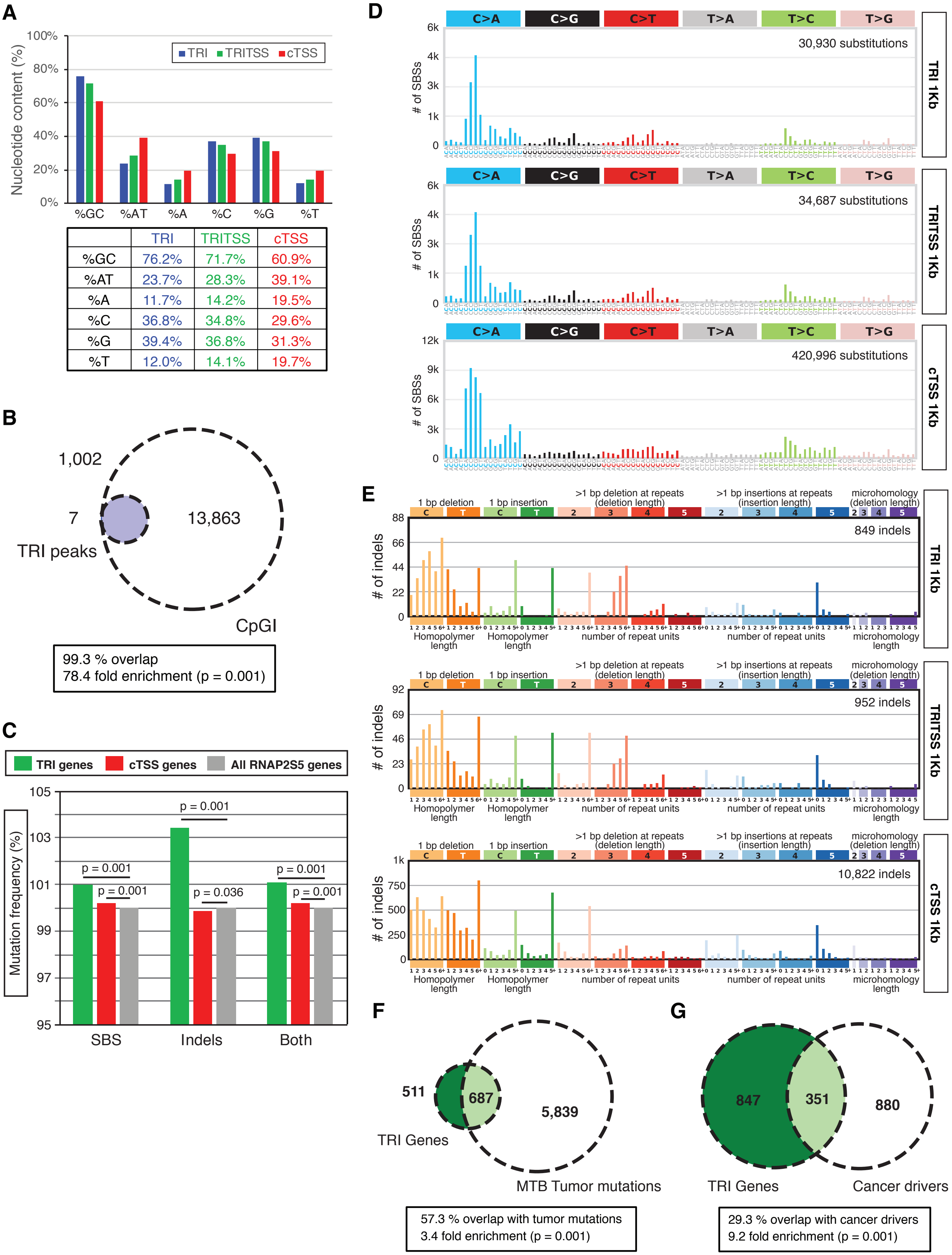

	TRI	TRITSS	cTSS
%GC	76.2%	71.7%	60.9%
%AT	23.7%	28.3%	39.1%
%A	11.7%	14.2%	19.5%
%C	36.8%	34.8%	29.6%
%G	39.4%	36.8%	31.3%
%T	12.0%	14.1%	19.7%
